# GBE1 Promotes Glioma Progression by Enhancing Aerobic Glycolysis through Inhibition of FBP1

**DOI:** 10.3390/cancers15051594

**Published:** 2023-03-03

**Authors:** Zhen Chen, Han Bao, Jingfang Long, Peiqi Zhao, Xiaowei Hu, Hao Wang, Ying Zhang, Jianjing Yang, Qichuan Zhuge, Lei Xia

**Affiliations:** 1 Zhejiang Provincial Key Laboratory of Aging and Neurological Disorder Research, The First Affiliated Hospital of Wenzhou Medical University, Wenzhou 325000, China; 2 Department of Neurosurgery, The First Affiliated Hospital of Wenzhou Medical University, Wenzhou 325000, China; 3 Central Laboratory, The First Affiliated Hospital of Wenzhou Medical University, Wenzhou 325000, China

**Keywords:** glucan branching enzyme 1, Warburg effect, glucose metabolism, fructose-bisphosphatase 1, NF-κB

## Abstract

**Simple Summary:**

Due to the poor prognosis of glioma patients and the limitations of glioma treatment, our study aimed to find new targets for glioma on metabolic therapy. Our study reveals a role for glycogen branching enzyme 1 (GBE1) in regulating glioma initiation and progression. We found that the expression of GBE1 correlated with a poor prognosis in glioma patients. Moreover, GBE1 promotes glioma progression by enhancing aerobic glycolysis through the inhibition of fructose–bisphosphatase 1 (FBP1), which reveals GBE1 as a potential target for glioma therapy.

**Abstract:**

Tumor metabolism characterized by aerobic glycolysis makes the Warburg effect a unique target for tumor therapy. Recent studies have found that glycogen branching enzyme 1 (GBE1) is involved in cancer progression. However, the study of GBE1 in gliomas is limited. We determined by bioinformatics analysis that GBE1 expression is elevated in gliomas and correlates with poor prognoses. In vitro experiments showed that GBE1 knockdown slows glioma cell proliferation, inhibits multiple biological behaviors, and alters glioma cell glycolytic capacity. Furthermore, GBE1 knockdown resulted in the inhibition of the NF-κB pathway as well as elevated expression of fructose-bisphosphatase 1 (FBP1). Further knockdown of elevated FBP1 reversed the inhibitory effect of GBE1 knockdown, restoring glycolytic reserve capacity. Furthermore, GBE1 knockdown suppressed xenograft tumor formation in vivo and conferred a significant survival benefit. Collectively, GBE1 reduces FBP1 expression through the NF-κB pathway, shifting the glucose metabolism pattern of glioma cells to glycolysis and enhancing the Warburg effect to drive glioma progression. These results suggest that GBE1 can be a novel target for glioma in metabolic therapy.

## 1. Introduction

Gliomas are the most common malignant tumors in the central nervous system and are divided into circumscribed gliomas and diffuse gliomas, according to WHO CNS5 in 2021 [1,2]. Glioblastoma (GBM), the most common and fatal diffuse glioma, accounts for 57.3% of gliomas [3]. Treatment of GBM is often unsatisfactory due to the limited extent of surgical resection and the presence of the blood-brain barrier (BBB) and the complex tumor microenvironment, with a median survival of fewer than two years and only 6.9% of patients who survive more than five years after diagnosis [4,5]. Therefore, studying the pathogenesis of glioma and finding the factors driving tumorigenesis and progression are essential for treating glioma.

The Warburg effect endows tumor cells with the ability to use aerobic glycolysis to meet their high–metabolite needs, but it also makes tumor metabolism a unique target for targeted therapy. Studies have found that the Warburg effect promotes tumor progression in multiple ways, including by reducing toxic metabolites [6,7], methylating tumor suppressor genes [8,9], and inhibiting immune responses [10,11,12]. Thus, targeting tumor metabolism may inhibit tumor progression from multiple aspects. A recent study found that inhibition of the basic leucine zipper and W2 domain 1 (BZW1) suppresses pancreatic cancer cell proliferation by inhibiting glycolysis under oxygen and glucose deprivation conditions [13]. Ovo Like Zinc Finger 2 (OVOL2), a transcription factor, inhibits the Warburg effect and breast cancer progression by suppressing the expression of glycolytic genes [14]. Moreover, in ovarian cancer, fibrillin-1 (FBN1) knockdown enhances cisplatin sensitivity by inhibiting glycolysis and angiogenesis [15]. Furthermore, the disruption of glycolysis in gliomas suppressed intracranial tumors and prolonged the median survival time of mice [16].

The loss of function of glycogen branching enzymes (GBE1) is the cause of glycogen metabolic disorders such as Glycogen Storage Disease IV (GSD–IV) and Adult Polyglucosan Body Disease (APBD) [17,18]. However, an increasing number of studies have shown its relevance to cancer. On the one hand, GBE1 is highly expressed in acute myeloid leukemia (AML) and maintains abnormal tumor cell proliferation by inhibiting AMPK activity [19]. GBE1 expression is also elevated in lung adenocarcinomas and is associated with worse survival [20]. On the other hand, GBE1 expression is decreased in ovarian cancer, and GBE1 downregulation is correlated with poor clinical outcomes [21]. These studies demonstrate that GBE1 plays different roles in different tumors. However, there has not been enough significant research on GBE1 in gliomas.

Herein, we evaluated GBE1’s role in gliomas, confirming that the expression of GBE1 is elevated in gliomas and correlates with a poor prognosis. We then demonstrated through cellular and animal experiments that GBE1 influences FBP1 expression through the NF-κB pathway, which affects the glucose metabolism pattern of glioma cells and promotes the Warburg effect to drive tumor progression. This study provides a potential target for glioma metabolic therapy.

## 2. Materials and Methods

### 2.1. Cell Culture

Human glioma cell lines U87, ln229, and U251; HEK–293t engineered cells, as well as human umbilical vein endothelial cells (HUVECs), were purchased from the Shanghai Institute of Biosciences and Cell Resources Center (Chinese Academy of Sciences, Shanghai, China) and cultured in Dulbecco’s Modified Eagle Medium (DMEM, Gibco, C11995500BT, Waltham, MA, USA) supplemented with 5% fetal bovine serum (FBS, Gibco, 16000044, Waltham, MA, USA) and 1% penicillin–streptomycin (Gibco, 15070063, Waltham, MA, USA) in an incubator at 37 °C, 5% CO_2_.

### 2.2. Plasmid Construction and Lentiviral Transfection

Plasmid construction and lentiviral transfection were performed as previously described [22,23]. Oligonucleotides targeting the following mRNA sequences were synthesized by Sangon Biotech (Shanghai, China) (sh-GBE1-1: AAAGGTAGTTATTACTAGTAAA, sh-GBE1-2: TTCGCTACAAGTTCCTAAATAA, and sh-FBP1: TACCAACGTGACAGGTGATCAA) and integrated into a lentiviral vector expressing mCherry fluorescent protein. oe-FBP1 and oe-NC plasmids were provided by Youze Bio (Guangzhou, China). Recombinant plasmids (sh-GBE1, sh-FBP1, and oe-FBP1) and empty plasmids (sh-NC and oe-FBP1) were co-transfected with packaging plasmids (pRSV-Rev, pMDLg pRRE, and VSV-G) into HEK-293t cells to produce lentiviral particles. Glioma cells were infected with the supernatant containing lentiviral particles for 24 h. Infected cells were screened by the BD FACSAria cell sorter (BD, Franklin Lakes, NJ, USA) according to the expression of mCherry.

### 2.3. RNA Extraction and Real−Time Quantitative PCR (qRT−PCR)

The total RNA of glioma cells was extracted by Trizol (Thermo Scientific, 15596018, Waltham, MA, USA), and then the cDNA libraries were constructed using the RevertAid RT reverse transcription kit (Thermo Scientific, k1691, Waltham, MA, USA). qRT-PCR was performed with the following program for 40 cycles: 95 °C/15 s, 60 °C/15 s, and 72 °C/45 s. The cycle threshold (CT) value of the target RNA was normalized to that of GAPDH. The relative expression was finally calculated by the 2-△△CT method. The qRT-PCR primer sequences were as follows: GBE1-Forward: 5′-GGACTTCCAGCGCAGGTATAA-3′, GBE1-Reverse: 5′-ATCAGCACATCTGTGGACGC-3′, FBP1-Forward: 5′-CCTACTGCCCTCTCTTGCCG-3′, FBP1-Reverse: 5′-CCATGACGAAGCGGGTCAG-3′, GAPDH-Forward: 5′-TGACATCAAGAAGGTGGTGAAGCAG-3′, GAPDH-Reverse: 5′-GTGTCGCTGTTGAAGTCAGAGGAG-3′.

### 2.4. Protein Extraction and Western Blot (WB)

The total protein of glioma cells was extracted with RIPA lysis solution (Thermo Scientific, 89900, Waltham, MA, USA) containing a protease phosphatase inhibitor cocktail (Beyotime, p1045, Shanghai, China) and phenylmethanesulfonyl fluoride (PMSF, Beyotime, p1045, Shanghai, China). Protein concentration was determined using a BCA protein assay kit (Thermo Scientific, 23227, Waltham, MA, USA). Protein samples were separated by sodium dodecyl sulfate-polyacrylamide gel electrophoresis (SDS-PAGE) and transferred to polyvinylidene fluoride membranes (PVDF, Merck Millipore, IPFL85R, Darmstadt, Germany). They were then blocked with a TBST (TBS, 0.1% Tween) solution containing 5% skimmed milk for 2 h at room temperature and incubated with primary antibodies overnight at 4 °C. Finally, they were incubated with horseradish peroxidase (HRP) conjugated secondary antibodies for 1 h at room temperature. Protein bands were developed using an ECL luminescence reagent (Meilunbio, MA0186, Dalian, China). Details of the antibodies are as follows: Anti-GBE1 (Abcam, ab180596, 1:1000, Cambridge, UK), Anti-FBP1 (Affinity, DF7325, 1:1000, Suyang, China), Anti-HIF1α (CST, 36169, 1:1000, Boston, MA, USA), Anti-MMP9 (Affinity, BF0560, 1:1000, Suyang, China), Anti-VEGFA (Abcam, ab46154, 1:1000, Cambridge, UK), Anti-Bcl-2 (Affinity, AF6139, 1:1000, Suyang, China), Anti-Bax (Affinity, AF0120, 1:1000, Suyang, China), Anti-Caspase-3 (Abcam, ab32351, 1:1000, Cambridge, UK), Anti-Cyclin D1 (Affinity, AF0931, 1:1000, Suyang, China), Anti-Cyclin D1 (Affinity, AF0931, 1:1000, Suyang, China), Anti-p65 (Affinity, AF5006, 1:1000, Suyang, China), Anti-Phospho-p65 (Affinity, AF2006, 1:1000, Suyang, China), Anti-GAPDH (Affinity, AF7021, 1:1000, Suyang, China), Anti-beta-Tubulin (Abcam, ab78078, 1:1000, Cambridge, UK), Goat Anti-Mouse IgG (Biosharp, BL001A, 1:5000, Hefei, China), Goat Anti-Rabbit IgG (Biosharp, BL003A, 1:5000, Hefei, China).

### 2.5. Cell Proliferation Assay and Colony Formation Assay

Cell proliferation assays were performed using Cell Counting Kit-8 (CCK-8, MCE, HY-K0301, Monmouth, NJ, USA). Briefly, a CCK-8 reagent was added to the culture medium according to the instructions, then placed in an incubator at 37 °C with 5% CO_2_ in the dark for 2 h. Finally, the absorbance at 450 nm was measured using a microplate reader.

U87 cells (1000 per well), ln229 cells (2000 per well), and U251 cells (2000 per well) were seeded in 6-cm dishes and then cultured in an incubator at 37 °C with 5% CO_2_. A fresh complete medium was replaced every 3 days. After 14 days of culture, cells were fixed with 4% paraformaldehyde (Solarbio, p1110, Beijing, China) and stained with 2.5% crystal violet (Meilunbio, MA0148, Dalian, China). Pictures were finally taken with a digital camera by a researcher who was blinded to the group allocation to calculate the colony formation rate. Colony formation rate = amount of colonies/number of seeded cells.

### 2.6. Cell Cycle Analysis and Apoptosis Detection

The cell cycle was detected using the DNA content quantification assay (Solarbio, CA1510, Beijing, China). Briefly, cells were fixed with 75% alcohol overnight at 4 °C after digestion with trypsin (Gibco, 25200072, Waltham, MA, USA). RNase A was used the next day to remove RNA. Then PI staining solution was added, and the cells were incubated for 30 min in the dark at 4 °C. The cell cycle was finally detected using Beckman Coulter Cytoflex (BeckmanCoulter, Brea, CA, USA).

Apoptosis was detected using an Annexin V-FITC apoptosis detection kit (Solarbio, CA1020, Beijing, China). Briefly, cells were digested with an EDTA-free trypsin (Gibco, 15050057, Waltham, MA, USA) and resuspended in a 1× binding buffer. FITC-labeled anti-Annexin V was added for 5 min at room temperature in the dark. Finally, the PI staining solution was added, and cell apoptosis was detected using Beckman Coulter Cytoflex (BeckmanCoulter, Brea, CA, USA).

### 2.7. Wound Healing Assay and Transwell Assay

Once 90% cell confluence was reached, a wound was created with a 200 μL pipette tip, and floating cells were washed. Fresh serum-free DMEM was added, and three locations were randomly selected and photographed under a microscope (Olympus, Tokyo, Japan) to record the initial wound area. The cells were then incubated at 37 °C with 5% CO2 to continue the culture. Photographs were taken at the same location every 12 h, and the area of scratch reduction was counted as the wound healing area. All the measurements were performed by a researcher who was blinded to group allocation.

Transwell chambers (Corning, 3422, New York, NY, USA) were used to further assess cell migration and invasion capabilities. For the migration assay, after 24 h of serum-free starvation, cells were resuspended in serum-free DMEM and seeded at a density of 2 × 10^5^/mL in the upper chamber and in the lower chambers with a complete medium containing 10% FBS. The chambers were then placed in a 37 °C, 5% CO_2_ incubator for another 24 h.

The transwell chambers were coated with Matrigel (Corning, 354234, New York, NY, USA) for the invasion assay. After cells were serum-free starved for 24 h, they were resuspended in serum-free DMEM and seeded at a density of 4 × 10^5^/mL in the upper chamber and the lower chamber with a complete medium containing 10% FBS. The chambers were then placed in a 37 °C, 5% CO_2_ incubator for 48 h.

Cells were fixed with 4% paraformaldehyde after culture, stained with crystal violet after scratching off the upper chamber cells, and finally photographed for counting under a microscope (Olympus, Japan, Tokyo, Japan) by a blinded researcher.

### 2.8. Oxygen Deprivation Assay and Tubule Formation Assay

Glioma cells (1 × 10^4^ per well) were seeded in 96 well plates and incubated at 37 °C in a 5% CO_2_ incubator for 24 h. The entire fresh medium was replaced before hypoxia. The plates were then incubated at 37 °C in a hypoxia incubator (5% CO_2_, 95% N_2_) for another 24 h, and cell proliferation was detected by CCK-8 reagent every 8 h.

U87 (1 × 10^7^ per dish), LN229 (5 × 10^6^ per dish), and U251 (5 × 10^6^ per dish) cells were seeded in 10 cm dishes. After 24 h of normal culture, cells were changed to serum-free DMEM and placed in a 37 °C hypoxia incubator (5% CO_2_, 95% N_2_) for another 24 h. The supernatant was collected and purified as conditioned medium (CdM). GFP-labeled HUVEC cells (8 × 10^4^ per well) were seeded in Matrigel-coated 96-well plates after resuspension with CdM. The cells were then placed in an incubator at 37 °C with 5% CO_2_ for 12 h, and pictures were taken under a fluorescence microscope (Leica, Wetzlar, Germany) every 3 h to record tubule formation. All measurements and observations of tubule formation assays were performed by an investigator blinded to group allocation.

### 2.9. Immunofluorescence Staining and Immunohistochemistry Staining

For immunofluorescence staining, glioma cells were blocked with 5% bovine serum albumin (BSA, Beyotime, ST023, Shanghai, China) in PBST solution (PBS, 0.4% Triton) for 1 h at room temperature after fixation with 4% paraformaldehyde. The primary antibodies were then added for overnight incubation at 4 °C. The next day, cells were incubated with fluorescence-conjugated secondary antibodies for 1 h at room temperature, and finally, the nuclei were stained with a DAPI staining solution (Solarbio, S2110, Beijing, China). Images were acquired using a fluorescence microscope (Leica, Wetzlar, Germany) by a blinded researcher.

For immunohistochemical staining, tumor and adjacent tissues from three glioblastoma patients were collected at the Department of Neurosurgery in the First Affiliated Hospital of Wenzhou Medical University. Tissue sections were dewaxed and hydrated and then soaked in 3% H_2_O_2_ for 10 min to remove endogenous catalase. The antigen was then repaired with a citrate-EDTA antigen recovery solution (Beyotime, P0086, Shanghai, China). Tissue sections were then blocked with 5% BSA in PBST for 1 h at room temperature and incubated with primary antibodies overnight at 4 °C. The following day, tissue sections were incubated with HRP-labeled secondary antibodies at 37 °C for 1 h and then developed with the DAB Color Development Kit (Beyotime, P0202, Shanghai, China) for 5 min. Finally, the nuclei were stained with hematoxylin (Beyotime, C0107, Shanghai, China). All measurements and observations were performed by a blinded investigator. The collection of human specimens was approved by the Ethics Committee of the First Affiliated Hospital of Wenzhou Medical University (Ethics number: KY2021-R129).

The antibody details are as follows: Anti-KI67 (Abcam, ab16667, 1:1000, Cambridge, UK), Anti-GBE1 (Abcam, ab180596, 1:1000, Cambridge, UK), Dylight 488, Donkey anti-rabbit IgG (EarthOx, E032221, 1:500, Millbrae, CA, USA), Goat anti-rabbit IgG (Biosharp, BL003A, 1:5000, Hefei, China).

### 2.10. Glycolytic Stress Test and Mitochondrial Stress Test

Glioma cells (2 × 10^4^ per well) were seeded in Seahorse XF 96-well culture plates (Agilent, 102601, Santa Clara, CA, USA) and incubated at 37 °C in a 5% CO_2_ incubator for 24 h.

For the glycolytic stress test, cells were changed to detection medium (XF base medium, 2 mM glutamine) the next day, and the change in extracellular acidification rate (ECAR) after the sequential addition of glucose, oligomycin, and 2-Deoxyglucose (2-DG) was detected with the Seahorse XF Pro analyzer (Agilent, Santa Clara, CA, USA).

For the mitochondrial stress test, cells were changed to detection medium (XF base medium, 1 mM sodium pyruvate, 2 mM glutamine, and 10 mM glucose) the next day. Then, the change in cellular oxygen consumption rate (OCR) after the sequential addition of oligomycin, FCCP, and rotenone/antimycin A was detected with a Seahorse XF Pro analyzer (Agilent, Santa Clara, CA, USA).

### 2.11. Intracranial Tumor Formation

A total of 10 BALB-c/nude male mice aged 4 to 6 weeks were purchased from the Shanghai Charles River Experimental Animal Limited Liability Company (Shanghai, China) and housed under specific pathogen-free conditions (SPF) (20–23 °C, 55–60% humidity) at the laboratory animal center, the First Affiliated Hospital of Wenzhou Medical University. Lentivirus (sh-NC or sh-GBE1) infected U87 cells carrying luciferase reporters were prepared. Nude mice were sequentially numbered and randomly divided into two groups (n = 5). Then, 5 × 10^5^ cells were transplanted into the striatum of nude mice according to the mouse brain anatomical atlas (https://atlas.brain-map.org, accessed on 5 May 2022). The optical density values of xenografts were measured every seven days using the IVIS in vivo optical imaging system (PerkinElmer, Waltham, MA, USA). The natural death time of nude mice was recorded for survival analysis. All measurements and observations were performed by a blinded researcher. All animal experiments were approved by the animal ethics committee of Wenzhou Medical University (Ethics number: WYYY-2021–0214).

### 2.12. Bioinformatics Analysis Based on Public Databases

We performed differential expression analysis and Kaplan–Meier survival analysis of GBE1 by gene expression profiling interactive analysis (GEPIA). To further analyze the association of GBE1 expression with glioma grade, IDH mutation, and 1p/19q co-deletion, we obtained RNA-seq and clinical data from 584 glioma patients from the Cancer Genome Atlas (TCGA), including 441 low-grade gliomas (LGG) and 143 glioblastomas. RNA expression profiles were presented as fragments per kilobase of exon model per million mapped fragments (FPKM). Moreover, we obtained the data of 686 glioma patients from the Chinese glioma Genome Atlas (CGGA) and did the same analysis for further demonstration. Finally, based on the TCGA database, ROC curves were generated according to the expression of GBE1 in LGG and GBM.

### 2.13. Statistical Analysis

All statistical analyses of this study were performed using GraphPad Prism 9. Student’s *t*-test was used to determine whether differences between two data groups conformed to the normal distribution, and variance analysis was used to compare data among multiple groups. The homogeneity of variance was tested by the Leneve test. Kaplan–Meier survival curves were compared using the Log-rank (Mantel–Cox) test. Data are presented as mean ± standard deviation (SD), and a two-tailed *p*-value < 0.05 was considered statistically significant.

## 3. Results

### 3.1. GBE1 Expression Was Associated with Glioma Malignancy

**Figure 1:**
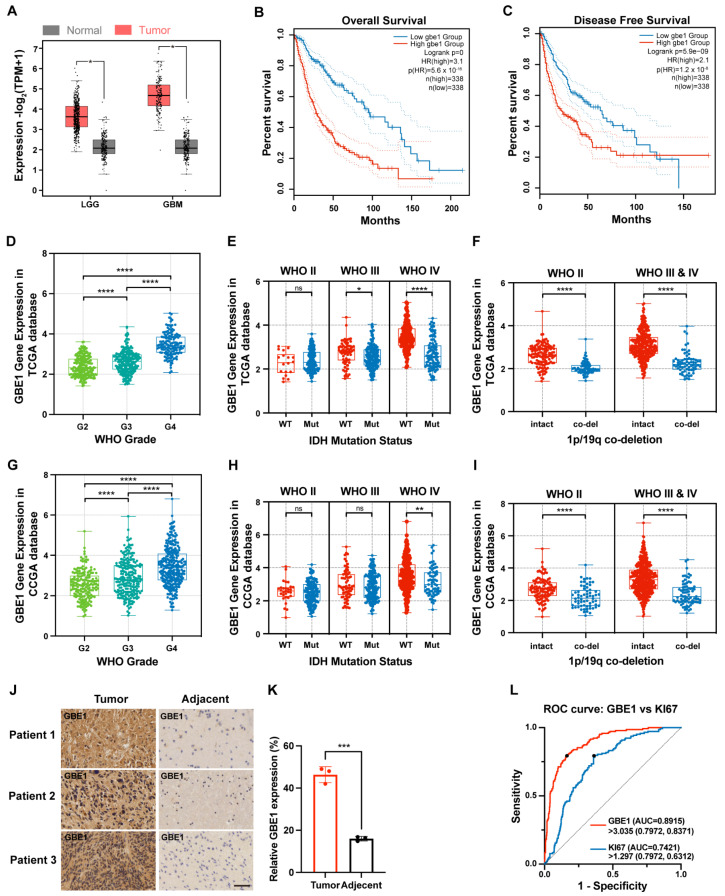
GBE1 expression was associated with the malignancy of gliomas. (**A**) Differential expression analysis of GBE1 in gliomas according to the GEPIA database (n-LGG = 518; n-GBM = 163; n-Normal = 207). (**B**) Overall Survival and (**C**) Disease-Free Survival of patients with GBE1-Low and GBE1-High gliomas according to the GEPIA database (n-low = 338; n-high = 338). GBE1 expression is classified by WHO grade, IDH mutation status, and 1p/19q co-deletion in the TCGA (n = 584) (**D**–**F**) and CGGA (n = 686) (**G**–**I**) databases. (**J**,**K**) Representative images of IHC staining for GBE1 in glioma specimens and adjacent normal specimens. Scale bar: 50 μm (below). (**L**) ROC curves for distinguishing glioma grade using GBE1 and Ki67 according to the TCGA (n = 584) database, GBE1: AUC = 0.8915; cut-off value = 3.035 (FPKM); sensitivity = 79.72%; specificity = 83.71%, KI67: AUC = 0.7421; cut-off value = 1.297 (FPKM); sensitivity = 79.72%; specificity = 63.12%. Data are presented as mean ± SD (ns *p* > 0.05; * *p* < 0.05; ** *p* < 0.01; *** *p* < 0.001; **** *p* < 0.0001).

GBE1 was overexpressed in a variety of tumors. To investigate GBE1 expression in gliomas, we performed a differential expression analysis through the GEPIA database (gepia2.cancer-pku.cn) and found that its expression was significantly higher in both LGG and GBM samples compared to normal samples (Figure 1A). Additionally, the overall survival and disease-free survival of patients in the GBE1 high-expression group were significantly lower than those in the low-expression group (Figure 1B,C). The mRNA expression profiles and clinical information of 584 glioma samples from the TCGA database and 686 glioma samples from the CGGA database revealed a significant positive correlation between GBE1 expression and WHO grade of gliomas (Figure 1D,G). Further, we subdivided the glioma samples according to IDH mutation status and 1p/19q deletion status and found higher GBE1 expression in IDH wild-type gliomas compared to IDH mutant ones (Figure 1E,H). As well as the higher expression of GBE1 in intact 1p/19q samples compared to co-deleted samples (Figure 1F,I). Furthermore, the results of IHC staining of tumor tissues and adjacent normal tissues from three glioma patients confirmed the higher expression of GBE1 in glioma samples (Figure 1J,K). These results suggested that GBE1 expression parallels the malignancy of gliomas. Subsequently, we evaluated the performance of GBE1 in distinguishing glioma grades by ROC curve. Its area under the curve (AUC) was 89.15% compared to 74.21% of KI67 (Figure 1L), indicating that GBE1 can be considered a discriminator of glioma malignancy.

### 3.2. GBE1 Knockdown Inhibited Glioma Cell Proliferation and Induced Cell Cycle Arrest and Apoptosis

**Figure 2:**
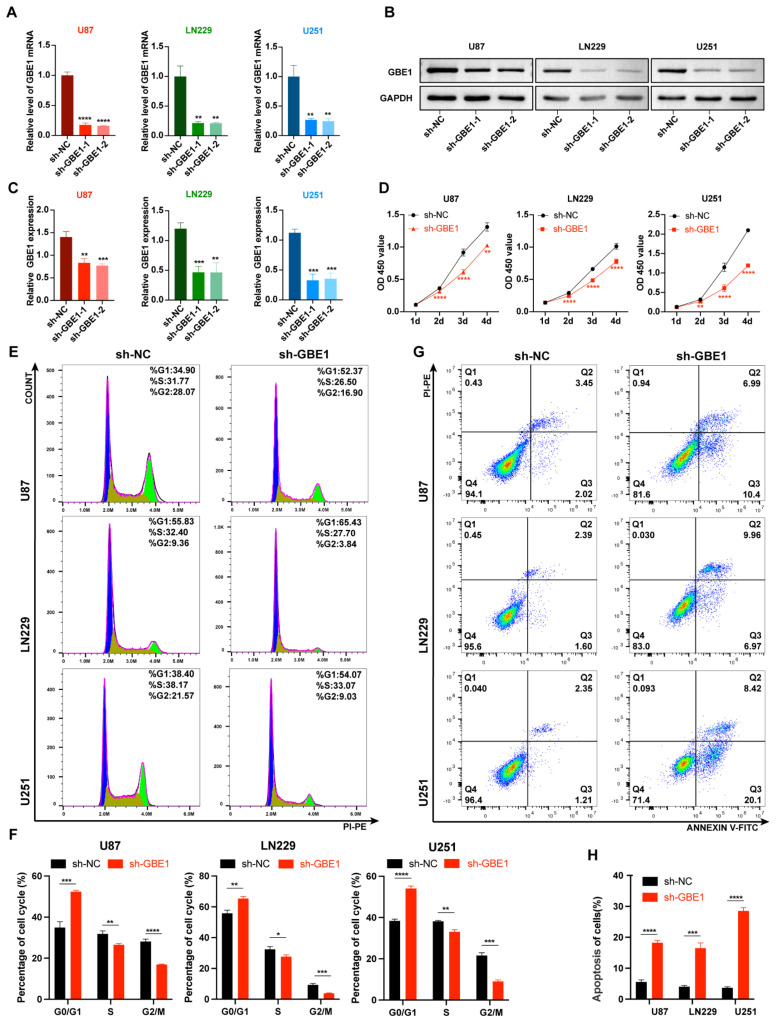
GBE1 knockdown inhibited glioma cell proliferation and induced cell cycle arrest and apoptosis. (**A**) The knockdown efficiency of two different lentiviral sequences analyzed by qRT-PCR in U87, LN229, and U251 cell lines (n = 3). (**B**,**C**) The knockdown efficiency of two different lentiviral sequences was analyzed by Western blot in three glioma cell lines (n = 3). Original blot see Supplementary Materials. (**D**) Cell viability of three glioma cell lines with GBE1 knockdown compared to the negative control. The OD 450 value was measured by the CCK-8 assay (n = 6). (**E**,**F**) Cell cycle analysis of three glioma cell lines with GBE1 knockdown compared to the negative control. Blue in the histogram represents the G0/G1 phase, yellow represents the S phase, and green represents the G2/M phase. (**G**,**H**) Apoptosis detection of the three glioma cell lines with GBE1 knockdown compared to the negative control. The sum of the Q2 and Q3 regions was considered the proportion of apoptotic cells. Cell cycle and apoptosis were detected by flow cytometry (n = 3). Data are presented as mean ± SD (* *p* < 0.05; ** *p* < 0.01; *** *p* < 0.001; **** *p* < 0.0001).

Bioinformatics analysis showed a strong association of GBE1 with glioma. To further investigate the role of GBE1 in gliomas, stable GBE1 knockdown and negative control U87, ln229, and U251 cells were constructed by sh-GBE1-1, sh-GBE1-2, and sh-NC lentiviruses, and the knockdown efficiency was verified by qPCR and WB (Figure 2A–C). Since sh-GBE1-1 and sh-GBE1-2 exhibited almost identical knockdown efficiencies, we selected sh-GBE1-1 for subsequent experiments. The cell counting kit-8 assay was then used to detect cell proliferation. The results showed that the proliferation of all three glioma cell lines was inhibited in the GBE1 knockdown group compared with the negative control (Figure 2D). Moreover, cell cycle analysis showed that GBE1 knockdown caused a decrease in cells at the G2/M phase while causing a significant increase in cells at the G0/G1 phase (Figure 2E,F), indicating that GBE1 knockdown arrested the cell cycle at the G0/G1 phase. Furthermore, the results of apoptosis analysis showed that GBE1 knockdown induced apoptosis in all three glioma cell lines (Figure 2G,H). These results indicate that GBE1 knockdown inhibits glioma cell proliferation, and this kind of inhibition results from cell cycle arrest coupled with increased apoptosis.

### 3.3. GBE1 Knockdown Affected Various Biological Behaviors of Glioma Cells

**Figure 3:**
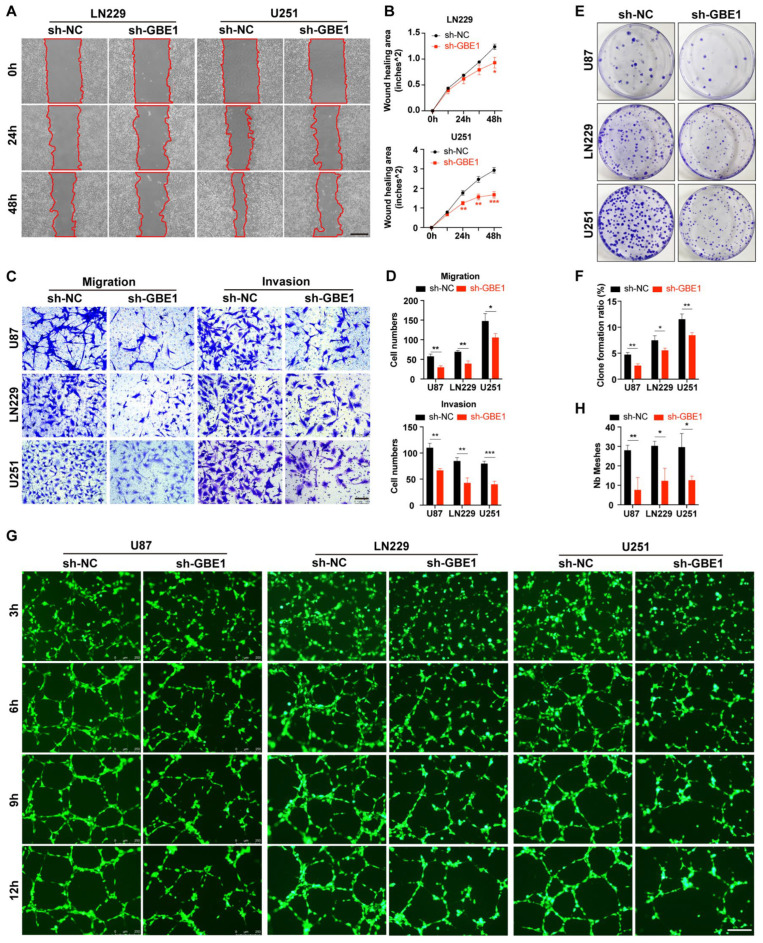
Knockdown of GBE1 affected various biological behaviors of glioma cells. (**A**,**B**) Wound healing assay of negative control and GBE1 knockdown ln229 and U251 cells. Photographs were taken every 12 h, and representative images at 0 h, 24 h, and 48 h were selected for presentation. The reduction area of the scratch was calculated as the wound healing area (n = 3). Scale bar: 500 μm. (**C**,**D**) Transwell assay of U87, ln229, and U251 cells of the negative control and GBE1 knockdown groups (n = 3). Five fields from top to bottom were selected for each chamber, and the average number of cells from three independent experiments was calculated. Scale bar: 100 μm. (**E**,**F**) Colony formation assay of three glioma cell lines transfected with sh-GBE1 compared with the negative control (n = 3). Colony formation ratio = amount of colonies/number of seeded cells. (**G**,**H**) Representative real-time images of tube formation assay. Human umbilical vein endothelial cells (HUVECs) were cultured with conditioned media collected from three glioma cell lines with GBE1 knockdown and a negative control (n = 3). Scale bar: 250 μm. Data are presented as mean ± SD (* *p* < 0.05; ** *p* < 0.01; *** *p* < 0.001).

To investigate whether GBE1 knockdown has additional effects on glioma, we assessed the alterations of glioma cells in biological behaviors such as migration, invasion, colony formation, and angiogenesis after GBE1 knockdown. First, a wound-healing assay was performed to assess the migration ability of glioma cells. Since the U87 cells grow in a grid-like pattern and it is difficult to form a dense cell monolayer, U251 cells and LN229 cells were selected for this assay. We found that the healing rate of ln229 and U251 cells was slowed after GBE1 knockdown compared with the negative control (Figure 3A,B). Since the wound healing assay cannot evaluate tumor invasion, a more comprehensive detection of invasion and migration is necessary. Therefore, we performed transwell assays using Matrigel to mimic the extracellular matrix. The results showed that the number of all three glioma cells that successfully crossed the transwell membrane was significantly decreased after GBE1 knockdown compared with the negative control, indicating that both the migration and invasion abilities of glioma cells were inhibited after GBE1 knockdown (Figure 3C,D).

Furthermore, the colony formation assay results showed that the colony formation ratios of all three glioma cell lines decreased significantly after GBE1 knockdown compared to the negative control (Figure 3E,F). Moreover, real-time recorded images of the tube formation assay (Figure 3G) showed that conditioned medium (CdM) from GBE1 knockdown glioma cells made HUVECs form fewer tubules compared with the negative control, indicating that GBE1 knockdown impaired the ability of glioma cells to promote angiogenesis (Figure 3H). GBE1 is involved in multiple biological behaviors of glioma cells, including migration, invasion, colony formation, and promotion of angiogenesis, and knockdown of GBE1 will impair these biological behaviors.

### 3.4. GBE1 Knockdown Affected the Biological Behavior of Glioma by Regulating Various Proteins and Affected the Expression of FBP1 through the NF−κB Pathway

**Figure 4:**
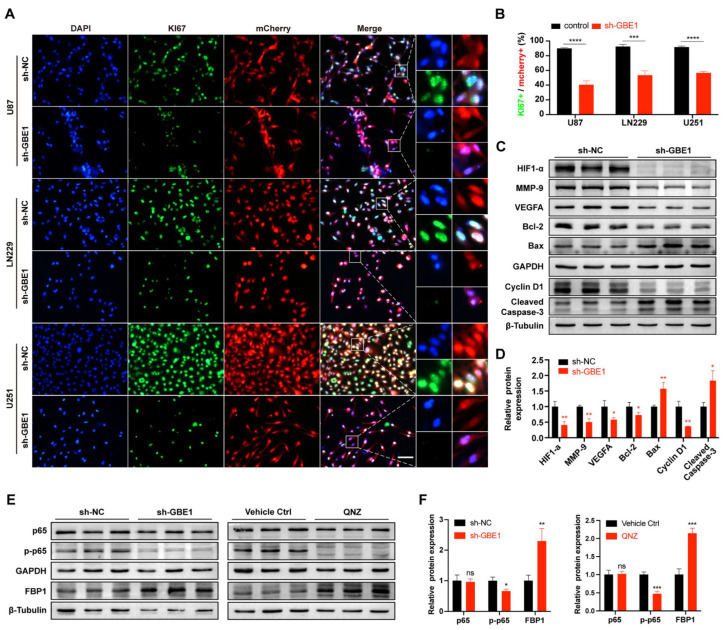
GBE1 knockdown affected the biological behavior of gliomas by regulating various proteins and affecting the expression of FBP1 through the NF-κB pathway. (**A**,**B**) Immunofluorescence staining of Ki67 in U87, ln229, and U251 cells (n = 3). Three fields from top to bottom were selected for each well, and the average of three independent wells was calculated. Scale bar: 100 μm. (**C**,**D**) Expression of tumor behavior-related proteins in GBE1 knockdown U251 cells compared with the negative control (n = 3). The protein levels of HIF1α, MMP-9, VEGFA, Bax, and Bcl-2 were normalized to GAPDH. Cyclin D1 and cleaved caspase-3 were normalized to β-Tubulin. (**E**,**F**) FBP1, total p65, and phosphorylated p65 protein levels in GBE1 knockdown U251 cells compared with single QNZ (1 μM) treatment (n = 3). Protein levels of total p65 and phosphorylated p65 were normalized to GAPDH, and FBP1 was normalized to β-Tubulin. Data are presented as mean ± SD (ns *p* > 0.05; * *p* < 0.05; ** *p* < 0.01; *** *p* < 0.001; **** *p* < 0.0001) Original blot see Supplementary Materials.

KI67 is strongly downregulated in resting G0 cells [24], making it a classic indicator of cell proliferation. The immunofluorescence staining of KI67 (Figure 4A) showed that after GBE1 knockdown, the resting cells with low KI67 expression in all three glioma cell lines increased significantly (Figure 4B), which was consistent with the results of cell cycle analysis. Since U251 cells exhibited more conspicuous and stable effects in most of the phenotypic experiments, including the CCK-8 assay, apoptosis detection, wound healing assay, and transwell invasion assay, this suggests that U251 cells were more vulnerable to GBE1 in cell proliferation, apoptosis, migration, invasion, and other classical phenotypes. Hence, we opted for U251 cells to further validate the expression of these phenotype-related proteins. We extracted the total protein of the GBE1 knockdown group and the negative control for western blot analysis. Cyclin D1 is a cell cycle-related protein whose reduced expression will lead to cell cycle arrest in the G1 phase [25,26]. Compared to the negative control, the expression of cyclin D1 was significantly reduced after GBE1 knockdown, which further proved that GBE1 knockdown could transform glioma cells into a resting state and inhibit proliferation (Figure 4C,D). The WB results also showed that GBE1 knockdown caused the downregulation of the invasion-related protein MMP9, the angiogenesis-related protein HIF1α, VEGFa, and the apoptosis inhibitor protein Bcl-2, and increased the expression of Bax and Cleaved-Caspase-3, further confirming the node role of GBE1 in the regulation of the biological behavior of glioma (Figure 4C,D).

GBE1 was found to cause promoter methylation of the FBP1 gene in lung adenocarcinoma cells through the NF-κB pathway, which decreased the expression of the FBP1 protein [27]. In glioma, we also observed that knockdown of GBE1 significantly inhibited the phosphorylation level of p65 protein, accompanied by elevated FBP1 expression (Figure 4E,F). To further investigate the relationship between GBE1, the NF-κB pathway, and FBP1, an NF-κB inhibitor was used to treat U251 cells to mimic NF-κB inhibition caused by GBE1 knockdown, and the expression of FBP1 after treatment was examined. QNZ, a neuroprotective inhibitor of the SOC channel, strongly inhibits NF-κB transcriptional activation [28]. The results of WB showed that QNZ (1 μM) decreased the level of phosphorylated p65 protein in U251 cells, while the total amount of p65 remained unchanged. And FBP1 levels were significantly elevated after QNZ treatment compared to vehicle control (Figure 4E,F), which paralleled the effect of GBE1 knockdown, indicating that the conclusion that GBE1 regulates FBP1 expression through the NF-κB pathway is also applicable in gliomas.

### 3.5. FBP1 Suppressed Malignant Phenotypes of Glioma Cells

**Figure 5:**
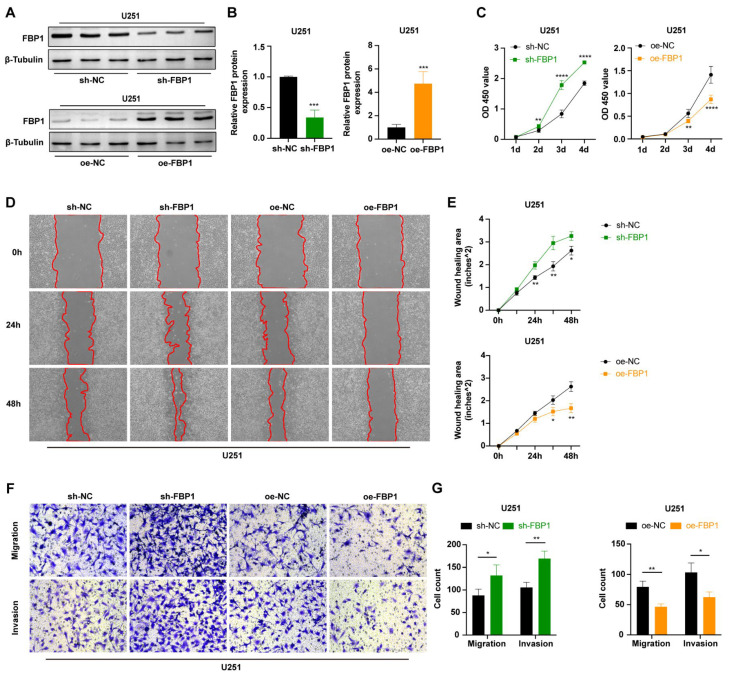
*FBP1*-suppressed malignant phenotypes of glioma cells. (**A**,**B**) The knockdown efficiency of the sh-*FBP*1 group and the overexpression efficiency of the oe-*FBP*1 group in U251 cells (n = 3) Original blot see Supplementary Materials. (**C**) Cellular viability of U251 cells in the FBP1 knockdown group and FBP1 overexpression group compared to the negative control. OD450 values were measured by the CCK-8 assay (n = 6). (**D**,**E**) Wound healing assay of FBP1 knockdown and FBP1 overexpression in U251 cells. Photographs were taken every 12 h, and representative images at 0 h, 24 h, and 48 h were selected for presentation. The reduced area of the scratch was calculated as the wound healing area (n = 3). Scale bar: 500 μM. (**F**,**G**) Transwell assay of FBP1 knockdown and FBP1 overexpression in U251 cells compared with the negative control (n = 3). Five fields from top to bottom were selected for each chamber, and the average number of cells from three independent experiments was calculated. Scale bar: 100 μm. Data are presented as mean ± SD (* *p* < 0.05; ** *p* < 0.01; *** *p* < 0.001; **** *p* < 0.0001).

As a tumor suppressor, FBP1 plays a critical role in the progression of multiple tumors [29,30,31]. Given that previous results showed GBE1 negatively regulated FBP1 expression in U251 cells (Figure 4E,F), to further verify the role of FBP1 in gliomas, we constructed U251 cells with FBP1 knockdown (sh-FBP1) and FBP1 overexpression (oe-FBP1). The knockdown and overexpression efficiencies were detected by Western blot (Figure 5A,B). The results of the CCK-8 assay showed that FBP1 knockdown accelerated U251 cell proliferation, while FBP1 overexpression inhibited the proliferation of U251 cells (Figure 5C). Additionally, wound healing assays showed that, compared to the sh-NC group, FBP1 knockdown significantly accelerated U251 cell migration and nearly closed the wound after 48 h migration. Conversely, compared to the oe-NC group, FBP1 overexpression significantly slowed the wound healing rate (Figure 5D,E). Further, transwell assays also showed that FBP1 knockdown significantly increased the number of cells that crossed the transwell membrane, enhancing U251 cell migration and invasion abilities, while FBP1 overexpression inhibited U251 cell migration and invasion (Figure 5F,G). These results indicate that FBP1 is an unfavorable factor for glioma cell proliferation, migration, and invasion. Increasing FBP1 expression helps to suppress these malignant phenotypes in glioma cells.

### 3.6. FBP1 Knockdown Reversed the Glioma Inhibition Caused by GBE1 Knockdown

**Figure 6:**
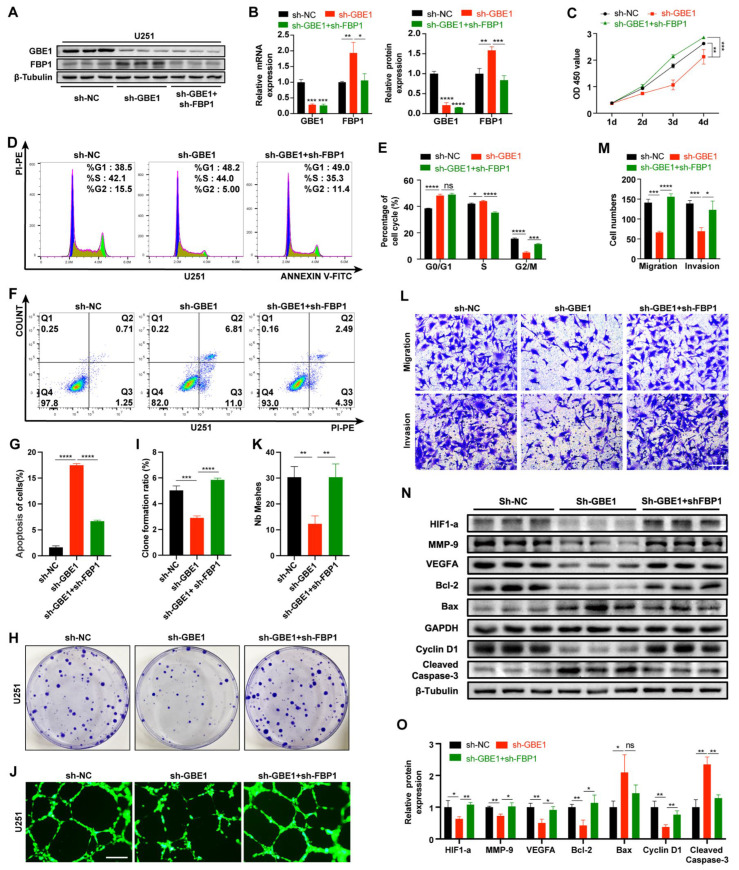
FBP1 knockdown reversed the glioma inhibition caused by GBE1 knockdown. (**A**,**B**) Knockdown efficiency of the sh-GBE1 group and sh-GBE1+shFBP1 group in U251 cells was detected by qRT-PCR (**Left B**) and Western blot (**Right B**) (n = 3) Original blot see Supplementary Materials. (**C**) The viability of U251 cells infected with sh-GBE1 or sh-GBE1 + sh-FBP1 was assessed by the CCK-8 assay (n = 6). (**D**–**G**) The effects of both GBE1 and FBP1 knockdowns on the cell cycle and apoptosis in U251 cells were assessed by flow cytometry (n = 3). (**H**–**M**) Effects of both GBE1 and FBP1 knockdown on cell colony forming ability, angiogenic ability, migration, and invasion ability of U251 cells; (**H**,**I**) colony forming ability was evaluated by colony formation assay (n = 3); colony formation ratio = amount of colonies/number of seeded cells. (**J**,**K**) Angiogenic capacity was assessed by tube formation assay, and representative images at 12 h were selected for presentation (n = 3); scale bar: 250 μm; (**L**,**M**) migration and invasion abilities were evaluated by transwell assay (n = 3); five fields from top to bottom were selected for each chamber, and the average number of cells from three independent experiments was calculated; scale bar: 100 μm. (**N**,**O**) Effects of knockdown of both GBE1 and FBP1 on the expression of proteins associated with tumor behavior in U251 cells (n = 3). Original blot see Supplementary Materials; data are presented as mean ± SD (ns *p* > 0.05; * *p* < 0.05; ** *p* < 0.01; *** *p* < 0.001; **** *p* < 0.0001).

To investigate whether GBE1 regulates glioma progression through FBP1, we constructed U87 and U251 cell lines in which both GBE1 and FBP1 were knocked down and observed whether the inhibition of glioma by single GBE1 knockdown could be reversed. The knockdown efficiency was verified by qPCR and WB, which showed that in the GBE1 and FBP1 knockdown group of U251 cells, the mRNA and protein content of FBP1 returned to the levels of the negative control (Figure 6A,B). In U87 cells, it was even lower than the negative control (Supplementary Figure S1A,B).

The results of the CCK-8 assay showed significantly enhanced cell viability in the group with knockdowns of both GBE1 and FBP1 compared with the single GBE1 knockdown group, even exceeding the negative control (Figure 6C and Supplementary Figure S1C). Meanwhile, the results of cell cycle analysis and apoptosis analysis revealed that the knockdown of FBP1 could reverse the cell cycle arrest and apoptosis caused by GBE1 knockdown, indicating that FBP1 knockdown significantly promoted tumor proliferation, demonstrating the powerful tumor suppressive effect of FBP1 (Figure 6D–G, Supplementary Figure S1D–G). Further studies revealed that glioma cells with knockdowns of both GBE1 and FBP1 showed a significant rebound in migration, invasion, vascularization, and colony formation ability compared with single GBE1 knockdowns (Figure 6H–M, Supplementary Figure S1H–M). Furthermore, the results of WB from U251 cells showed that changes in proteins related to tumor biological behaviors caused by single GBE1 knockdown were also reversed after FBP1 knockdown, which was paralleled to the alterations in tumor phenotypes (Figure 6N,O). These results suggest that the glioma suppressive effect caused by GBE1 knockdown is associated with increased expression of FBP1, and that knockdown of FBP1 will greatly impair this effect.

### 3.7. Elevated FBP1 Expression Caused by GBE1 Knockdown Induced Metabolic Reprogramming of Glioma Cells

**Figure 7:**
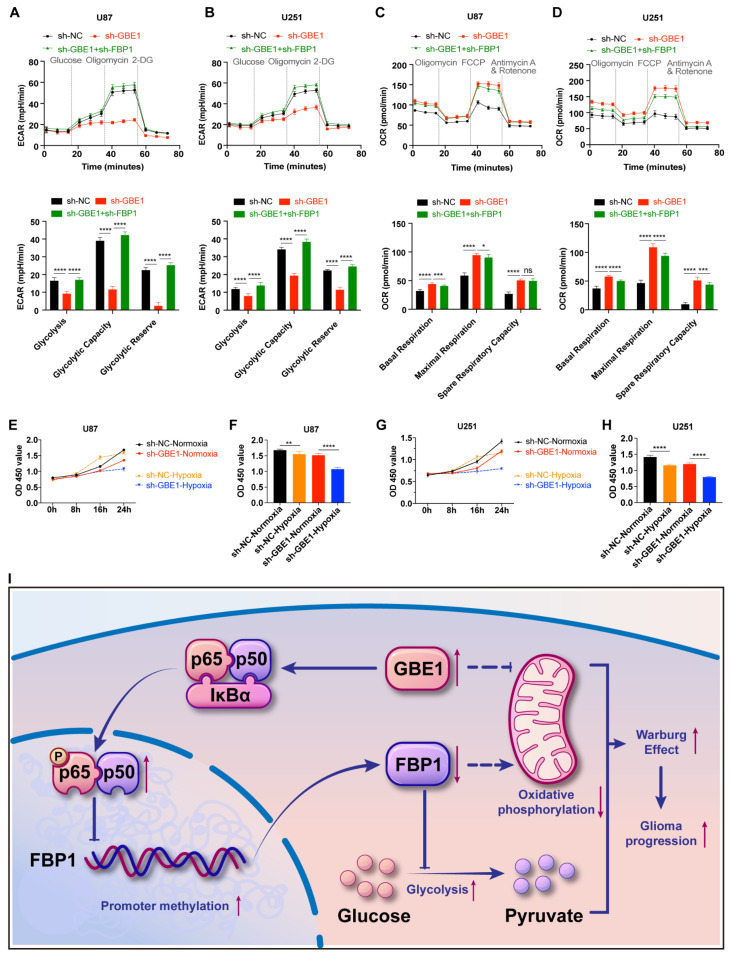
Elevated FBP1 expression caused by GBE1 knockdown induces metabolic reprogramming of glioma cells. (**A**,**B**) Extracellular acidification rate (ECAR) of negative control, sh-GBE1, and sh-GBE1 + sh-FBP1 in U87 and U251 cells. The effects of GBE1 and FBP1 on basal glycolysis, glycolytic capacity, and glycolytic reserve in glioma cells were assessed by ECAR. (**C**,**D**) Oxygen consumption rate (OCR) of negative control, sh-GBE1, as well as sh-GBE1 + sh-FBP1, in U87 and U251 cells. OCR reflects basal respiration, maximal respiration, and the spare respiratory capacity of glioma cells. Both ECARs and OCRs were detected by the Agilent Seahorse metabolic assay (n = 11). (**E**,**G**) Effect of GBE1 knockdown on the viability of glioma cells under hypoxia. (**F**,**H**) Cell viability at 24 h of hypoxia was selected for statistical analysis; cell viability was detected by the CCK-8 assay (n = 6). (**I**) The mechanism of GBE1 regulating the Warburg effect in glioma cells. Data are presented as mean ± SD (ns *p* > 0.05; * *p* < 0.05; ** *p* < 0.01; *** *p* < 0.001; **** *p* < 0.0001).

To further investigate why FBP1 inhibited glioma progression, considering the important role of FBP1 in glucose metabolism, we speculated that the glucose metabolic system of glioma cells was affected after GBE1 knockdown, which in turn inhibited glioma progression. Herein, we examined the extracellular acidification rate (ECAR), which reflects the glycolysis level of cells, using the Agilent Seahorse cell metabolic analysis system. The results showed that the basal glycolysis level and the glycolytic reserve capacity of U87 and U251 cells were inhibited after GBE1 knockdown. However, the knockdown of FBP1 reversed the inhibition of glycolysis caused by GBE1 knockdown, indicating that the increased expression of FBP1 is an important reason for the impaired glycolysis of glioma cells caused by GBE1 knockdown (Figure 7A,B). In addition, to evaluate the respiratory capacity of mitochondria, we also detected the cellular oxygen consumption rate (OCR). The results showed that GBE1 knockdown increased the basal respiration level and spare respiration capacity of mitochondria in both glioma cell lines. However, FBP1 knockdown had a limited effect on mitochondrial respiratory function, suggesting that other substances in addition to FBP1 are involved in the elevated mitochondrial respiration caused by GBE1 knockdown (Figure 7C,D).

To further verify the alteration of the metabolic pattern of glioma cells caused by GBE1 knockdown, we performed oxygen deprivation assays in U87 and U251 cells. According to the results of the CCK-8 assay, after 24 h of hypoxia, the viability of U87 cells in the control group decreased by 7.74%, whereas that of U87 cells in the GBE1 knockdown group decreased by 29.15%. Additionally, the viability of U251 cells in the control group decreased by 18.25%, compared to a 33.79% decrease in the viability of U251 cells in the GBE1 knockdown group. These results showed that after GBE1 knockdown, the tolerance of glioma cells to an anoxic environment was significantly reduced, which reflected the transformation of cellular metabolic patterns from glycolysis to oxidative phosphorylation after GBE1 knockdown (Figure 7E–H). These results indicate that GBE1 knockdown changes the metabolic mode of glioma cells from glycolysis to mitochondrial oxidative phosphorylation, and increased expression of FBP1 plays an important role in weakening the glycolysis level of glioma cells.

### 3.8. GBE1 Knockdown Significantly Inhibited the Growth of Glioma Xenograft In Vivo

**Figure 8:**
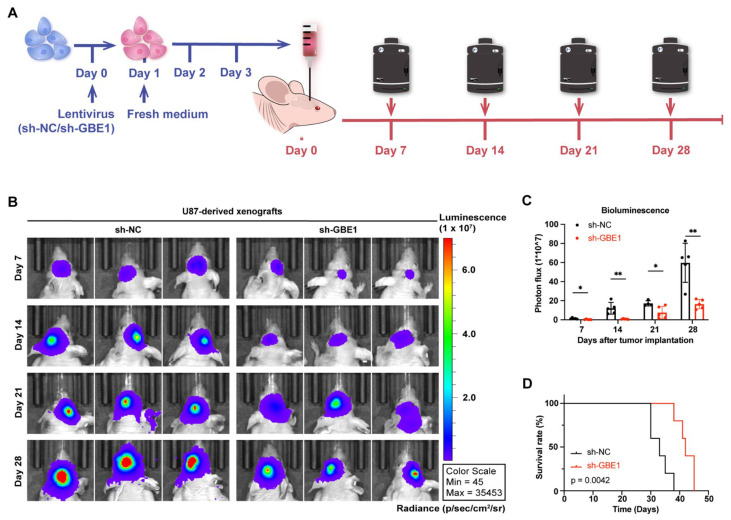
Knockdown of GBE1 significantly inhibited the growth of a glioma xenograft in vivo. (**A**) Schematic representation of intracranial tumor implantation and in vivo bioluminescence imaging by the IVIS in vivo imaging system. (**B**,**C**) Bioluminescence images and quantification of U87-derived xenografts in the brains of nude mice after GBE1 knockdown compared to the negative control (n = 5). (**D**) Kaplan–Meier survival analysis of nude mice after transplantation of GBE1 knockdown U87-derived xenografts compared with the negative control (n = 5). Data are presented as mean ± SD (* *p* < 0.05; ** *p* < 0.01).

To further investigate the effect of GBE1 knockdown on the growth of glioma cells in vivo, U87 cells carrying a luciferase reporter were transplanted into the striatum of nude mice at day four after sh-GBE1 or sh-NC lentivirus infection. The optical density values of xenografts were recorded every seven days by IVIS in vivo optical imaging system measurements (Figure 8A). The results of the bioluminescence imaging showed that the elimination of GBE1 significantly inhibited the growth of U87-derived xenografts (Figure 8B,C) and conferred a significant survival benefit compared to negative controls (Figure 8D). These results demonstrated that GBE1 knockdown effectively inhibited glioma growth in vivo and improved animal survival.

## 4. Discussion

Due to the heterogeneity of tumor cells, targeting molecules are not expressed in all tumor cells, making targeting therapies impaired and often unsatisfactory. However, metabolic abnormalities are a common feature of tumor cells [32], making targeting tumor metabolism a different idea for treating glioma. Our study found elevated expression of GBE1 in gliomas and a correlation with a poor prognosis. Furthermore, our findings validated the mechanism by which GBE1 affects glucose metabolism patterns for the first time in glioma (Figure 7I).

As one of the causes of glycogen metabolic diseases, recent studies have revealed that GBE1 affects the development and progression of a variety of tumors, such as leukemia, lung adenocarcinoma, and ovarian cancer [19,20,21]. However, the role of GBE1 in glioma is still unclear. Therefore, we analyzed TCGA and CGGA data and found that GBE1 expression was elevated in gliomas and correlated with a poor prognosis. Furthermore, GBE1 was more reliable than KI67 in predicting glioma grade. This was demonstrated when using respective cut-off values for both indexes; GBE1 showed a 20.59% higher specificity than KI67, although both had a sensitivity of 79.72%. In vitro, we found increased apoptosis, arrested cell cycle, and suppressed cell proliferation in glioma cells with GBE1 knockdown. Additionally, GBE1 knockdown also affected glioma cell migration, invasion, colony formation, and angiogenesis abilities. The impairment of these biological behaviors greatly affected the progression of glioma.

FBP1, the rate-limiting enzyme of gluconeogenesis, is generally considered a tumor suppressor and shows decreased expression in various tumors, such as renal, prostate, liver, and breast cancer [33,34,35,36]. In lung adenocarcinomas, GBE1 methylates the promoter of FBP1 through the NF-κB pathway and therefore decreases FBP1 expression [27]. Our results showed that the NF-κB pathway was inhibited following GBE1 knockdown in glioma cells, accompanied by elevated FBP1 expression; this paralleled the effects of single NF-κB pathway inhibitors, suggesting that regulation of FBP1 expression by GBE1 via NF-κB is also applicable in glioma. Despite the inhibitory effects of FBP1 on various tumors, it has also been shown that reduced FBP1 expression inhibits ovarian cancer formation and cisplatin resistance in vivo [37]. Furthermore, FBP1 promotes the proliferation, migration, and invasion of esophageal cancer cells by regulating fatty acid metabolism [38]. This suggests that the effects of FBP1 on tumors are multifaceted. Our study confirmed that in glioma cells, FBP1 expression was significantly elevated after GBE1 knockdown.

Recently, a study by Son B et al. found that radiation-induced downregulation of FBP1 expression promotes GBM cell migration [39]. Our study found that knockdown of FBP1 promoted the proliferation, migration, and invasion of glioma cells, while overexpression of FBP1 in glioma cells significantly inhibited the malignant phenotype of glioma cells, which paralleled the previous study. In contrast, knockdown of the elevated FBP1 based on GBE1 knockdown restored glioma cell proliferation and impaired tumor biological behavior. The inhibition of glioma caused by GBE1 knockdown was reversed. The above results illustrated that NF-κB pathway inhibition by GBE1 knockdown resulted in elevated FBP1, leading to glioma suppression.

The Warburg effect, characterized by aerobic glycolysis, promotes cancer progression [40] and generates many glycolytic intermediates that satisfy multiple anabolic reactions in cells [41]. Based on these theories, many studies targeting aerobic glycolysis have shown good tumor suppressive effects [13,14,15,16], exhibiting the potential of targeting tumor metabolism in tumor therapy. Our study found that FBP1 elevation by GBE1 knockdown suppressed the basal glycolytic rate and the glycolytic reserve capacity of glioma cells; this parallels a previous study on lung adenocarcinoma [27]. Hypoxia is one of the characteristics of the tumor microenvironment. GBE1 knockdown glioma cells in a hypoxic environment exhibit a malfunction in converting oxidative phosphorylation to glycolysis and a decreased tolerance to a hypoxic environment.

However, unlike lung adenocarcinoma, GBE1 knockdown increased oxidative phosphorylation in glioma cells and was difficult to reverse with FBP1 knockdown. This phenomenon has also been found in colon cancer and melanoma. Knockdown of glucose-6-phosphate isomerase caused glycolytic inhibition in colon cancer and melanoma cells while elevating the level of oxidative phosphorylation, which investigators consider to be a compensatory response of cells to glycolytic inhibition [42]. However, studies have found a direct inhibition of fructose-1,6-bisphosphate (F16bp), a substrate of FBP1, in rat liver mitochondria [43,44]. Although this explains the elevated oxidative phosphorylation caused by GBE1 knockdown, it cannot explain the inability to reverse this elevation by the knockdown of FBP1. Therefore, further investigation is needed to determine whether the increased level of oxidative phosphorylation caused by GBE1 knockdown is a compensatory response of cells to the inhibition of glycolysis or the influence of other factors. However, it is undeniable that the elevation of oxidative phosphorylation increases the dependency of glioma cells on oxygen and impairs their tolerance to hypoxia, which is beneficial for the treatment of glioma. Furthermore, the intracranial tumor formation assay also validated the inhibitory effect of GBE1 knockdown in a complex tumor microenvironment. Additionally, GBE1 knockdown conferred a significant survival benefit compared to controls.

## 5. Conclusions

Our study found that GBE1 expression was significantly elevated in gliomas and was correlated with a poor prognosis. GBE1 downregulates FBP1 expression through the NF-κB pathway, causing a shift in the glucose metabolism pattern of glioma cells to glycolysis, enhancing the Warburg effect, and promoting the development of glioma. These findings provide a new potential target for glioma treatment in the metabolic field.

## Data Availability

The data that support the findings of this study are available from the corresponding author upon reasonable request.

## Supplementary Materials

The following supporting information can be downloaded at: https://www.mdpi.com/article/10.3390/cancers15051594/s1, Figure S1: FBP1 knockdown in U87 cells reversed the tumor suppression caused by GBE1 knockdown. Figure S2: Original western blots.
