# Supplementary material for: GBE1 Promotes Glioma Progression by Enhancing Aerobic Glycolysis through Inhibition of FBP1
Source: Cancers (Basel). 2023 Mar 3;15(5):1594. doi: 10.3390/cancers15051594 (PMC10000543; doi:10.3390/cancers15051594)
Supplement: Supplementary file 1 [file cancers-15-01594-s001.zip › Supplementary Figure S1.pdf]

Supplementary Figures

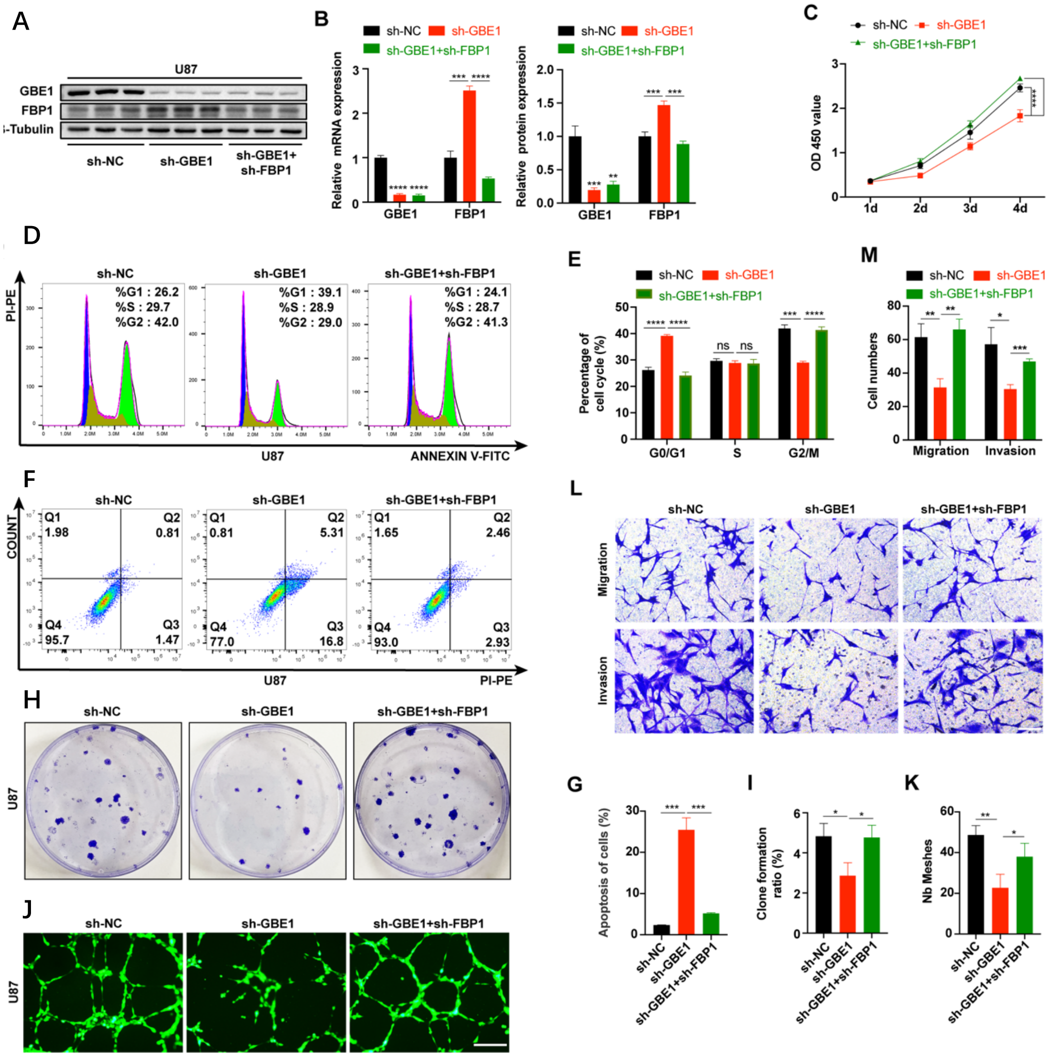

Figure S1. FBP1 knockdown in U87 cells reversed the tumor suppression caused by GBE1 knockdown. **(A, B)** Knockdown efficiency of sh-GBE1 and sh-GBE1 + sh-FBP1 in U87 cells (n = 3). **(C)** The viability of U87 cells infected with sh-GBE1 or sh-GBE1 + sh-FBP1 (n = 6). **(D, E)** Cell cycle analysis and **(F, G)** apoptosis analysis of U87 cells infected with sh-GBE1 or sh-GBE1 + sh-FBP1 (n = 3). **(H, I)** Colony formation ability, **(J, K)** angiogenic ability, **(L, M)** migration and invasion ability of U87 cells with both GBE1 and FBP1 knockdown; colony forming ability was evaluated by colony formation assay (n = 3); colony formation ratio = amount of colonies/number of seeded cells; angiogenic capacity was assessed by tube formation assay and representative images at 12 h were selected for presentation (n = 3); scale bar: 250 μm; migration and invasion abilities were evaluated by Transwell assay (n = 3); five fields from top to bottom were selected for each chamber, and the average number of cells from three independent experiments was calculated. Scale bar: 100 μm. Data are presented as mean ± SD (ns P > 0.05; *P < 0.05; **P < 0.01; ***P < 0.001; ****P < 0.0001).
