# Supplementary material for: GBE1 Promotes Glioma Progression by Enhancing Aerobic Glycolysis through Inhibition of FBP1
Source: Cancers (Basel). 2023 Mar 3;15(5):1594. doi: 10.3390/cancers15051594 (PMC10000543; doi:10.3390/cancers15051594)
Supplement: Supplementary file 1 [file cancers-15-01594-s001.zip › Supplementary Figure S2.pdf]

Figure.2B

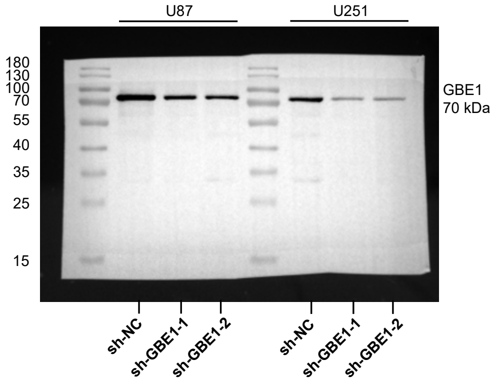

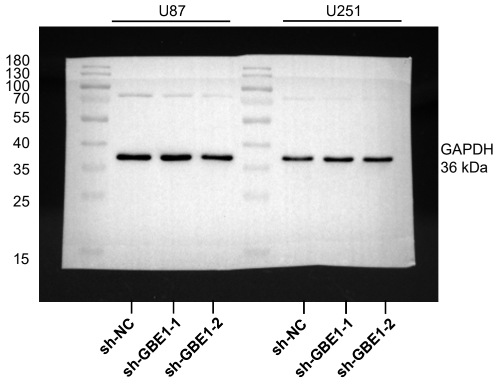

Line	U87 sh-NC	U87 sh-GBE-1	U87 sh-GBE-2	U251 sh-NC	U251 sh-GBE-1	U251 sh-GBE-2
GBE1	47795.401	21391.095	28152.338	28727.53	9711.874	8417.589
GAPDH	37589.874	29432.024	34571.874	24693.752	26972.681	25732.489

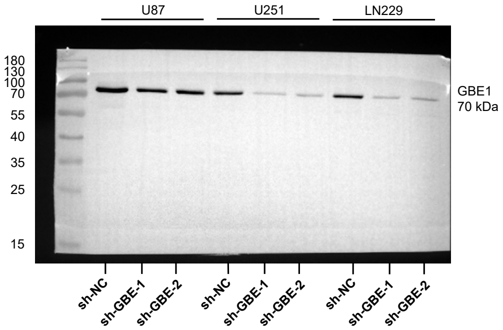

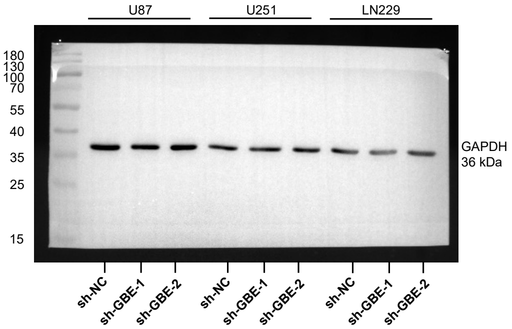

Line	U87 sh-NC	U87 sh-GBE-1	U87 sh-GBE-2	U251 sh-NC	U251 sh-GBE-1	U251 sh-GBE-2	LN229 sh-NC	LN229 sh-GBE-1	LN229 sh-GBE-2
GBE1	48174.693	29799.551	27791.773	38051.087	8200.853	9405.388	37215.894	9590.439	9762.631
GAPDH	31944.258	33816.359	37603.702	36099.087	38178.037	9405.388	29620.844	21353.187	25775.409

Figure.4C

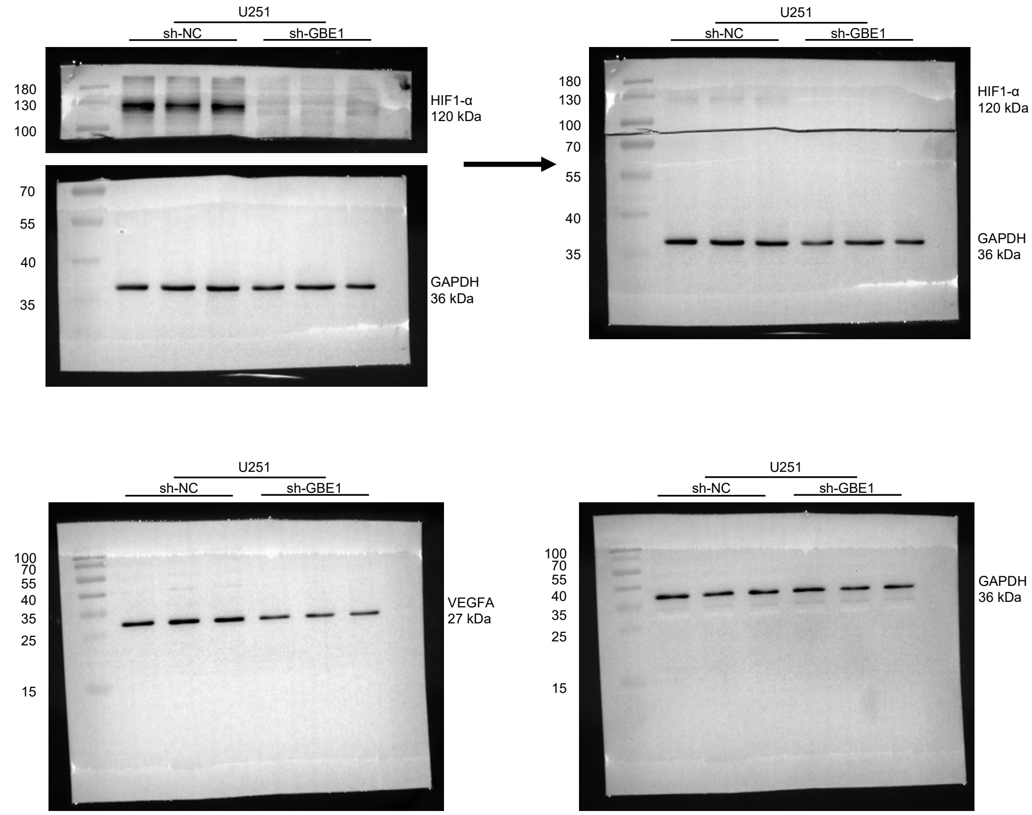

Line	U251 sh-NC	U251 sh-NC	U251 sh-NC	U251 sh-GBE1	U251 sh-GBE1	U251 sh-GBE1
HIF1-a	24261.782	22373.439	23069.317	7569.811	8055.447	9738.175
GAPDH	21147.246	25525.56	26559.681	20686.66	26623.731	18992.296
VEGFA	19664.024	23162.439	21047.489	13282.296	12702.589	11751.175
GAPDH	20399.267	16904.51	20227.752	22911.489	17539.711	17792.125

Figure.4C

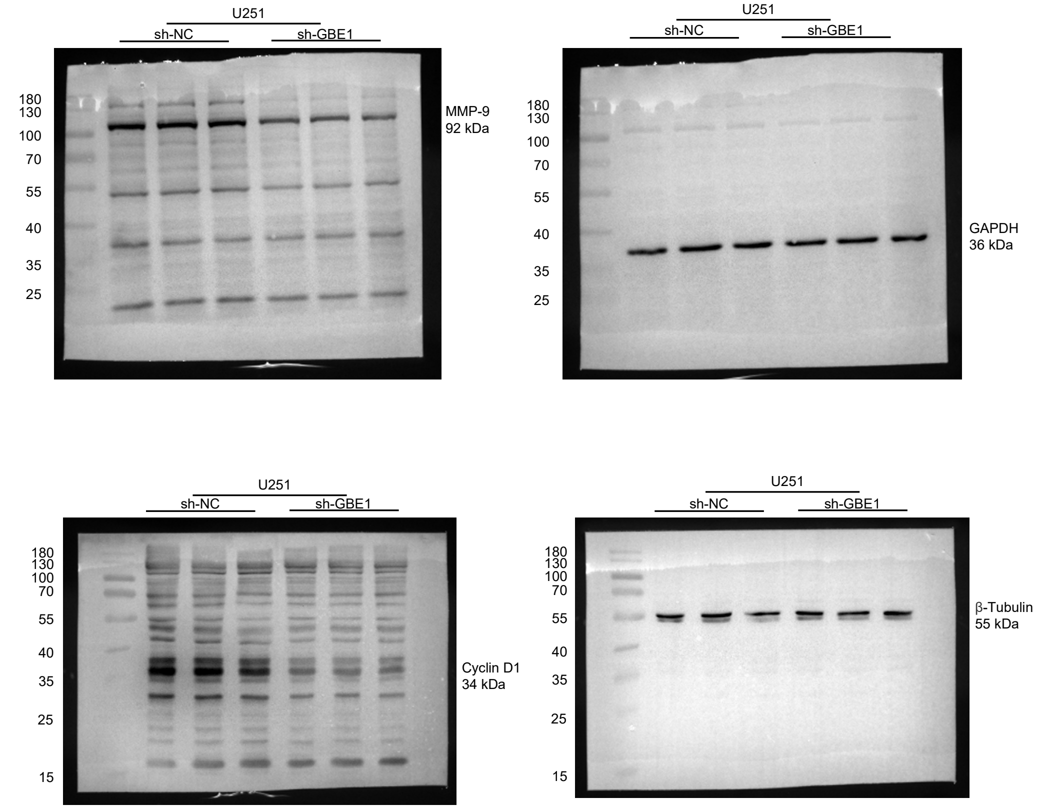

Line	U251 sh-NC	U251 sh-NC	U251 sh-NC	U251 sh-GBE1	U251 sh-GBE1	U251 sh-GBE1
MMP-9	136876.978	140336.635	121645.271	73466.907	60344.907	62236.149
GAPDH	40573.116	45568.016	38536.48	36446.823	41635.309	44456.652
Cyclin D1	23905.782	23363.004	19434.196	8575.69	8085.175	8316.811
β-Tubulin	18393.317	24054.368	19836.903	21563.418	20771.832	20783.882

Figure.4C

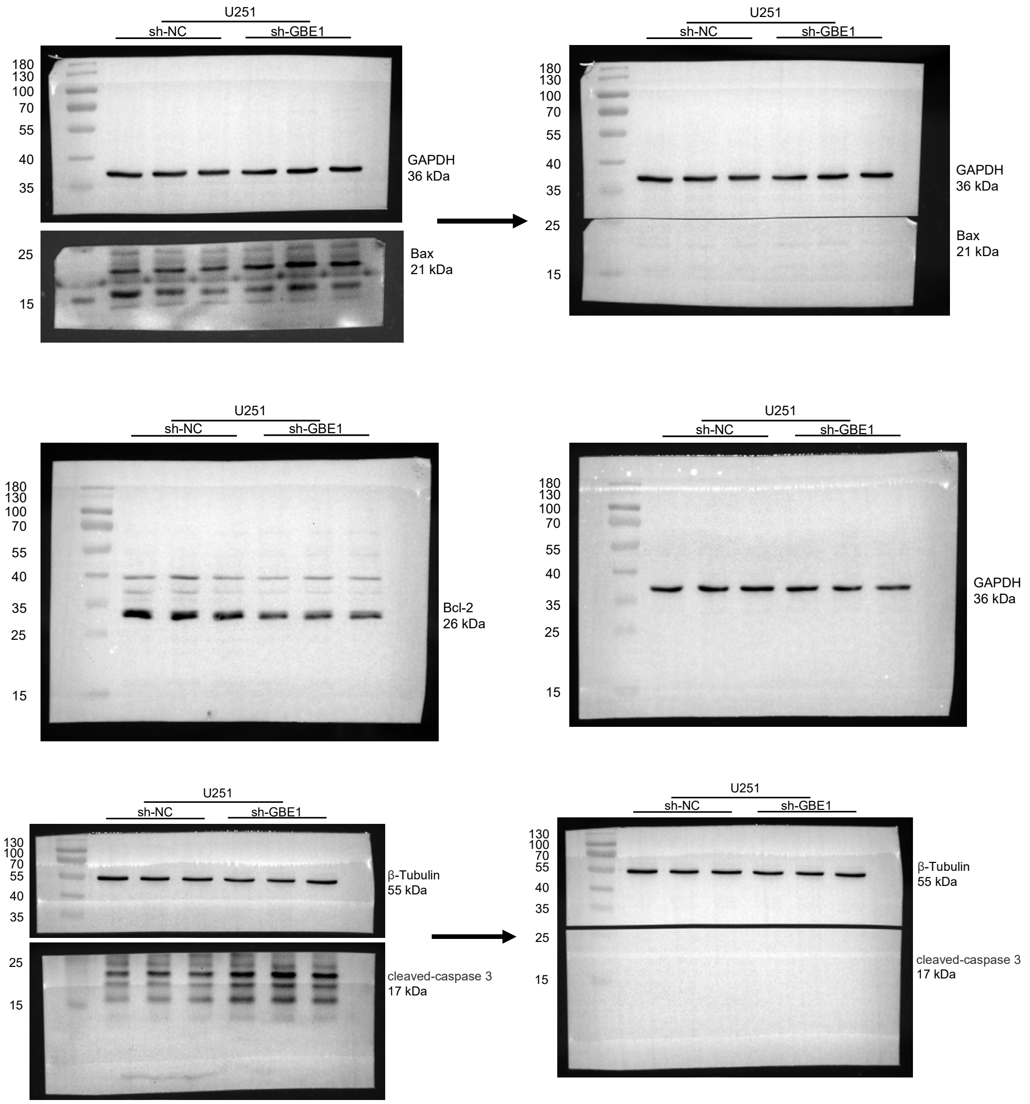

Line	U251 sh-NC	U251 sh-NC	U251 sh-NC	U251 sh-GBE1	U251 sh-GBE1	U251 sh-GBE1
Bax	136876.978	140336.635	121645.271	73466.907	60344.907	62236.149
GAPDH	40573.116	45568.016	38536.48	36446.823	41635.309	44456.652
Bcl-2	23905.782	23363.004	19434.196	8575.69	8085.175	8316.811
GAPDH	18393.317	24054.368	19836.903	21563.418	20771.832	20783.882
Cleaved-caspase 3	42804.652	45524.409	44719.258	66763.016	75467.238	70220.208
β-Tubulin	87307.794	74552.329	71169.572	63500.673	60758.53	80389.037

Figure.4E

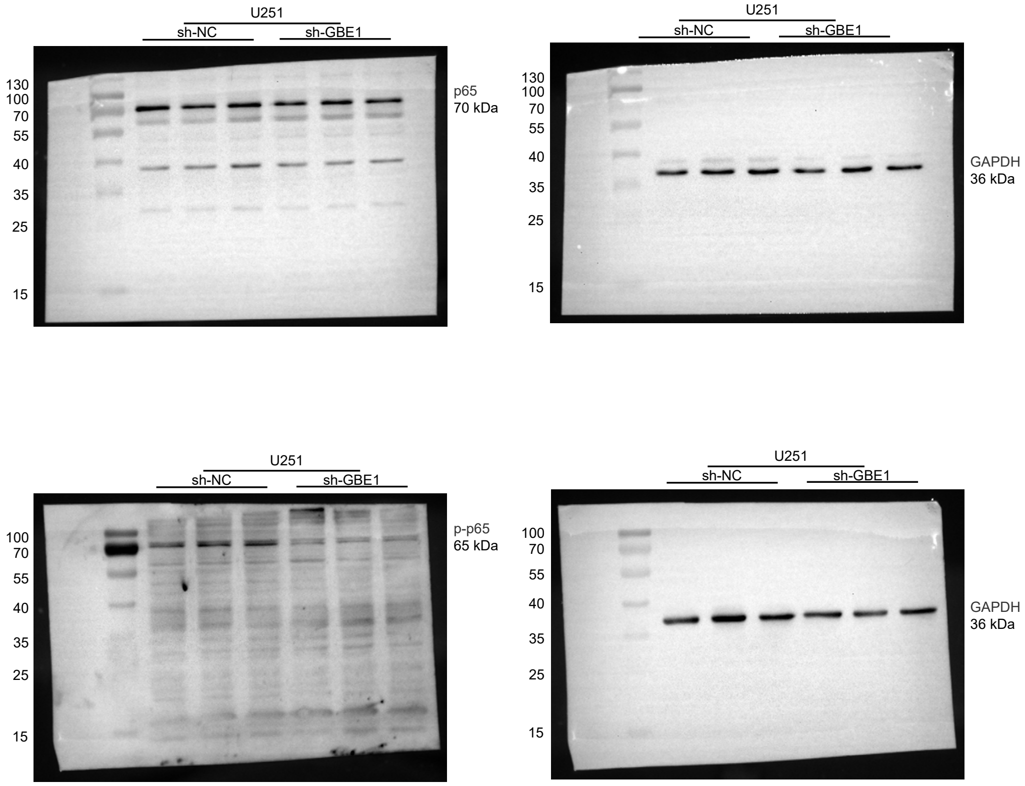

Line	U251 sh-NC	U251 sh-NC	U251 sh-NC	U251 sh-GBE1	U251 sh-GBE1	U251 sh-GBE1
P65	50969.72	43612.6	58148.97	46669.62	49053.89	46816.99
GAPDH	29420.82	36703.17	39889.6	29826.72	37994.17	34929.48
p-p65	60021.92	71264.33	71259.62	38247.38	30737.38	33168.38
GAPDH	54667.25	81718.28	67378.18	55630.18	42310.69	54770.47

Figure.4E

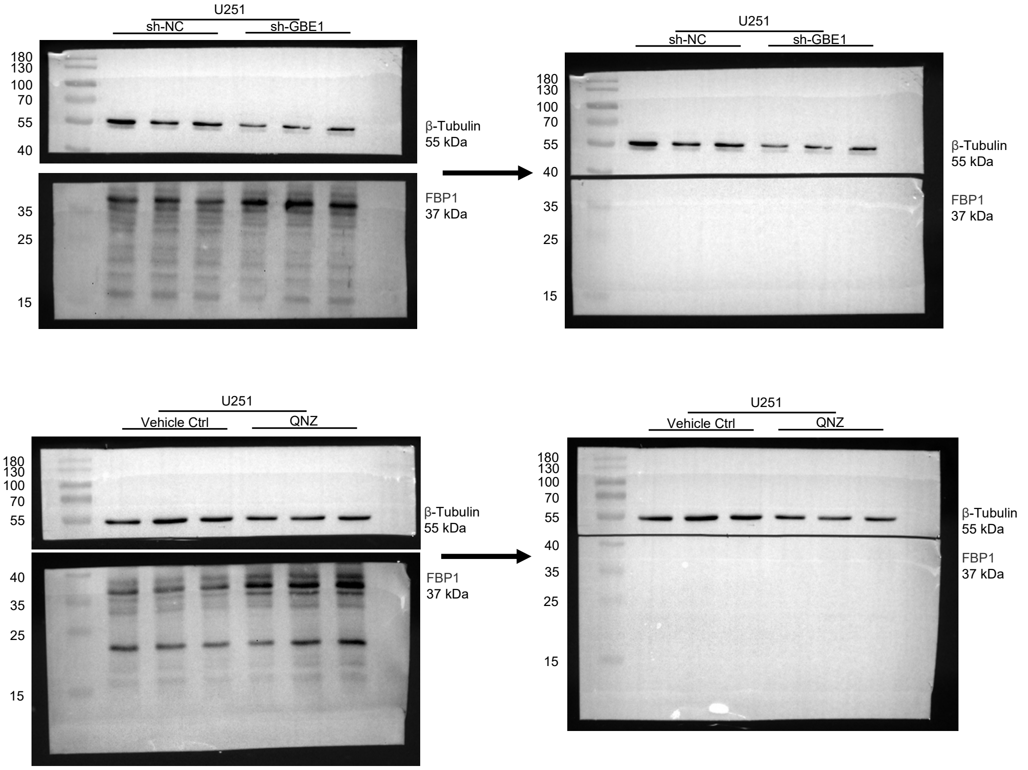

Line	U251 sh-NC	U251 sh-NC	U251 sh-NC	U251 sh-GBE1	U251 sh- GBE1	U251 sh- GBE1
FBP1	81020.241	67495.927	85599.392	106994.756	119733.141	107647.291
β-Tubulin	21394.024	12912.296	16060.196	8908.225	9800.054	12279.589

Line	U251 Vehicle Ctrl	U251 Vehicle Ctrl	U251 Vehicle Ctrl	U251 QNZ	U251 QNZ	U251 QNZ
FBP1	40249.844	37199.087	37924.43	60361.543	73635.614	76614.894
β-Tubulin	69133.371	88290.614	73528.936	56789.765	63206.543	74308.622

Figure.4E

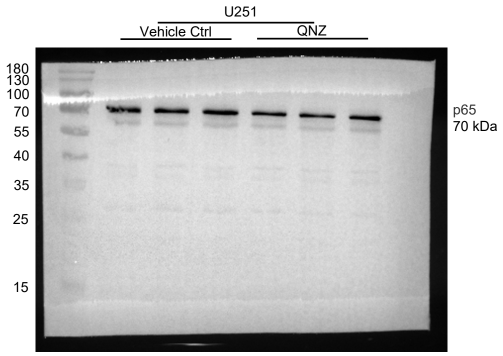

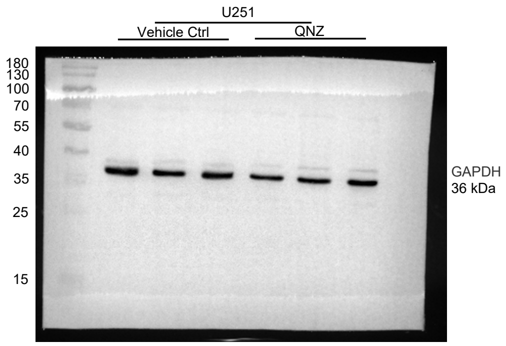

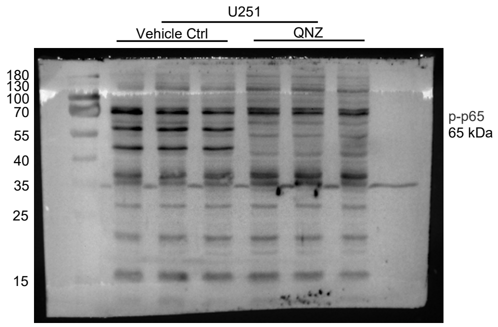

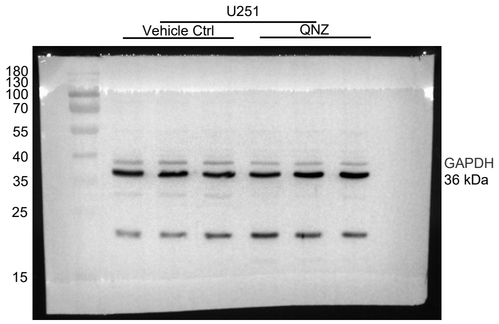

Line	U251 Vehicle Ctrl	U251 Vehicle Ctrl	U251 Vehicle Ctrl	U251 QNZ	U251 QNZ	U251 QNZ
P65	77645.643	74760.007	80976.007	63173.522	59906.765	69598.865
GAPDH	80419.472	63033.593	66642.765	51321.179	55810.714	61955.108
p-p65	63978.329	76545.3	67236.3	35811.3	32058.543	30755.966
GAPDH	73070.057	76662.3	75233.179	70397.229	78868.836	78060.057

Figure.5A

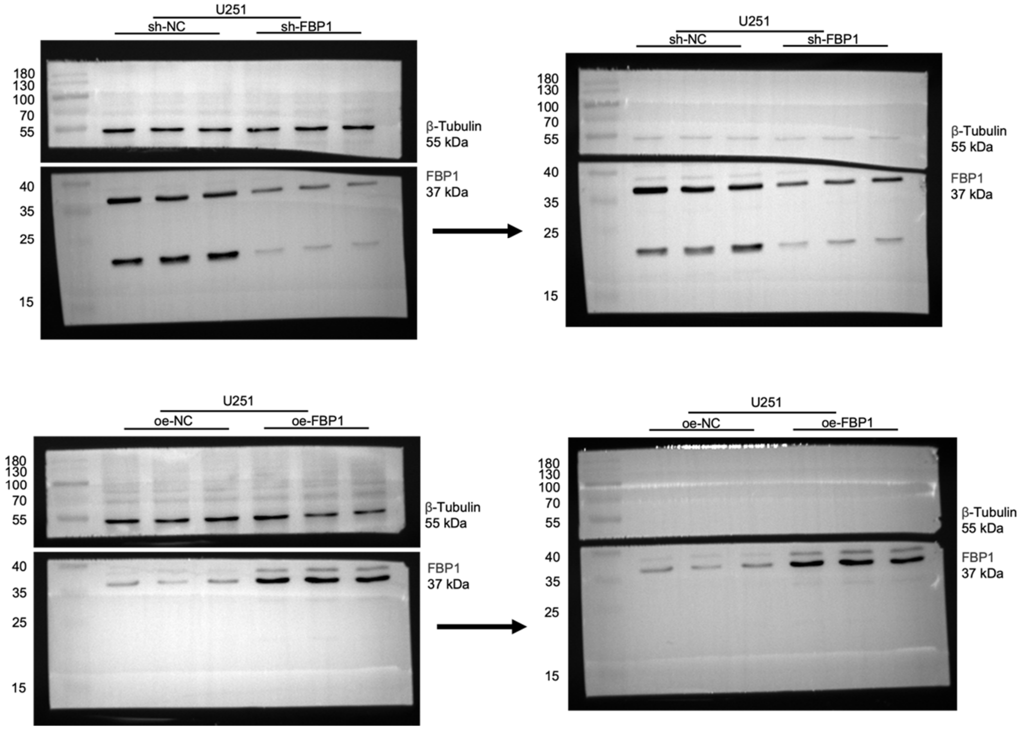

Line	U251 sh-NC	U251 sh-NC	U251 sh-NC	U251 sh-FBP1	U251 sh-FBP1	U251 sh-FBP1
FBP1	24811.125	20422.518	20092.276	10477.426	9593.012	7068.962
β-Tubulin	62937.643	53102.492	51904.765	58746.128	71700.38	82818.622

Line	U251 oe-NC	U251 oe-NC	U251 oe-NC	U251 oe-FBP1	U251 oe-FBP1	U251 oe-FBP1
FBP1	19605.581	9625.974	17243.702	67320.643	84230.957	94967.815
β-Tubulin	72615.522	60892.25	75297.492	86516.229	72555.279	80705.844

Figure.6A

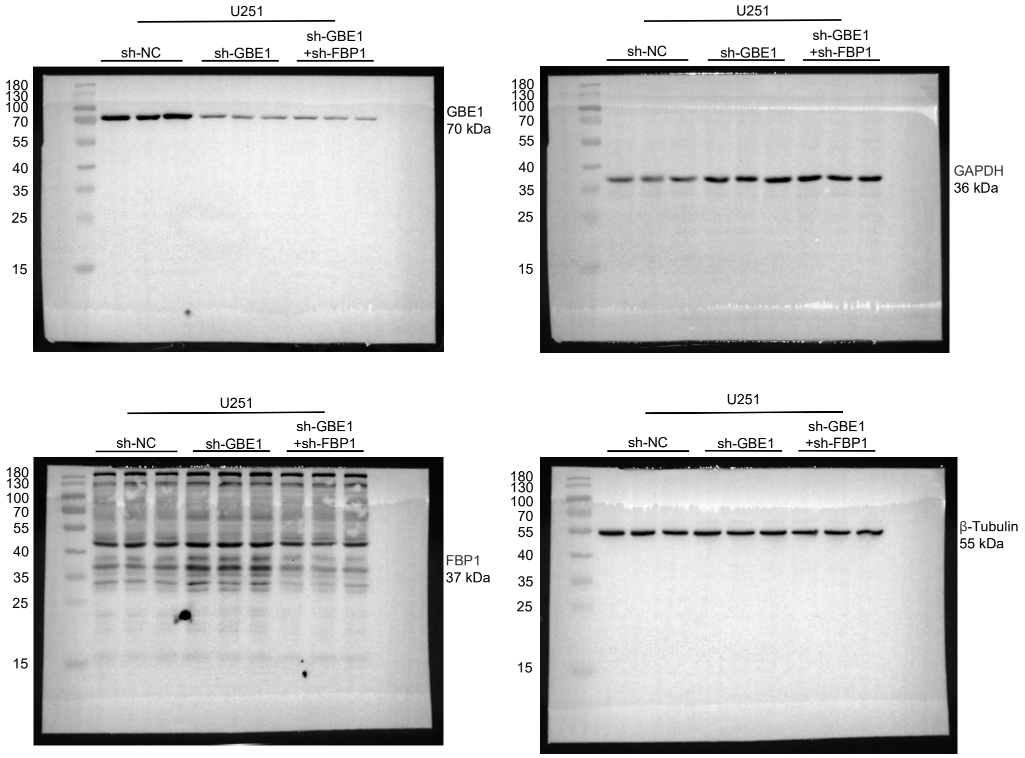

Line	U251 sh-NC	U251 sh-NC	U251 sh-NC	U251 sh-GBE1	U251 sh-GBE1	U251 sh-GBE1	U251 sh-GBE1 +sh-FBP1	U251 sh-GBE1 +sh-FBP1	U251 sh-GBE1 +sh-FBP1
GBE1	20513.74	16066.79	19593.569	6689.79	4713.719	4470.033	4209.255	3669.669	3780.79
GAPDH	12410.154	10296.083	11156.326	14621.326	13687.79	17337.447	16030.205	14698.083	14733.912
FBP1	10806.033	9005.012	11615.79	17009.497	16139.326	14905.083	8487.669	7466.548	7369.841
β-Tubulin	17511.033	15070.083	15342.669	16154.912	14759.497	15238.205	13475.79	13926.619	15226.033

Figure.S1A

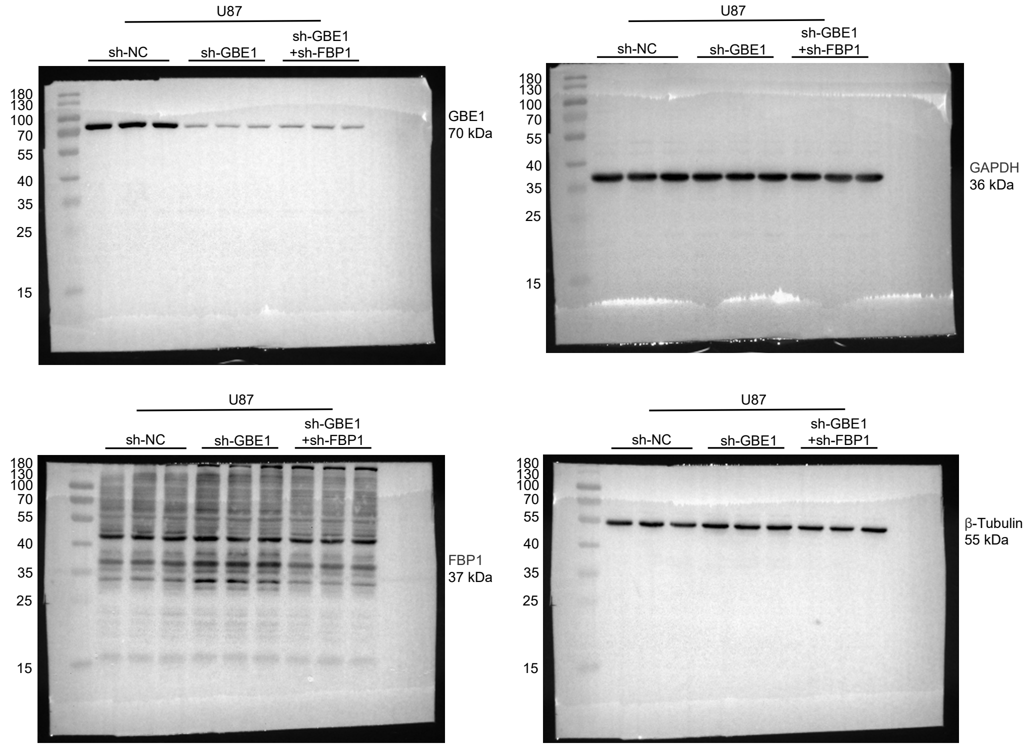

Line	U87 sh-NC	U87 sh-NC	U87 sh-NC	U87 sh-GBE1	U87 sh-GBE1	U87 sh-GBE1	U87 sh-GBE1 +sh-FBP1	U87 sh-GBE1 +sh-FBP1	U87 sh-GBE1 +sh-FBP1
GBE1	19252.205	18091.569	15050.74	4019.548	3146.497	2812.962	3506.861	4103.962	3938.891
GAPDH	19427.447	16202.619	18572.326	17787.033	17137.79	17149.669	16179.912	13738.79	13216.497
FBP1	10379.619	11263.255	11758.79	17470.74	17872.962	17081.619	10931.376	10549.376	10612.376
β-Tubulin	14876.861	14644.376	14746.083	16487.962	15602.669	15174.083	15834.255	15384.669	16718.619

Figure.6N

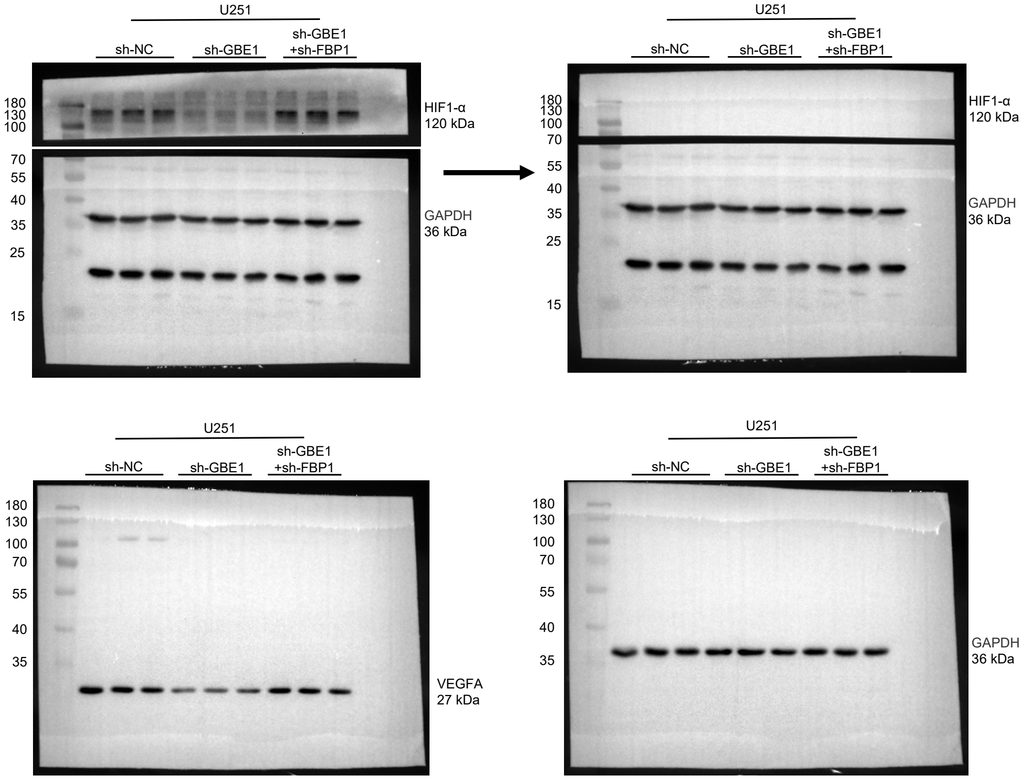

Line	U251 sh-NC	U251 sh-NC	U251 sh-NC	U251 sh-GBE1	U251 sh-GBE1	U251 sh-GBE1	U251 sh-GBE1 +sh-FBP1	U251 sh-GBE1 +sh-FBP1	U251 sh-GBE1 +sh-FBP1
HIF-1-α	51102.752	56174.652	57987.945	31581.045	26515.267	29629.631	55962.702	58294.581	48604.338
GAPDH	69780.53	54642.874	51918.095	48992.338	49679.288	45776.924	53991.752	52990.803	50035.823
VEGFA	63470.24	49025.46	41418.05	22661.46	23215.82	27120.05	44070.22	42178.63	36482.51
GAPDH	63554.12	56902.75	52984.05	59355.41	61028.7	47949.99	51013.87	49633.51	52311.58

Figure.6N

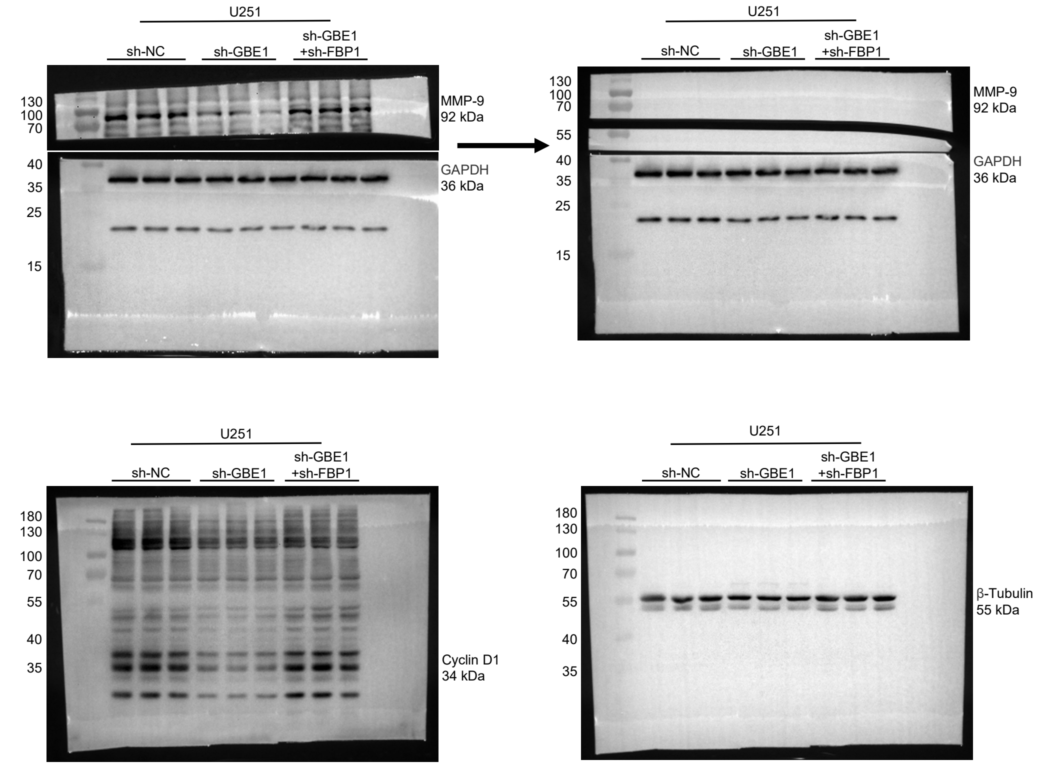

Line	U251 sh-NC	U251 sh-NC	U251 sh-NC	U251 sh-GBE1	U251 sh-GBE1	U251 sh-GBE1	U251 sh-GBE1 +sh-FBP1	U251 sh-GBE1 +sh-FBP1	U251 sh-GBE1 +sh-FBP1
MMP-9	58212.459	54605.53	46156.459	33070.723	33050.016	40191.652	49650.409	50413.823	45373.924
GAPDH	75934.288	68647.116	61814.388	63275.874	61073.995	65646.995	61676.288	58100.116	65419.723
Cyclin D1	61200.02	62932.58	52211.58	24895.05	17175.22	24605.99	49753.87	54604.63	40755.15
β-Tubulin	58147.41	46642.8	55682.51	53521.29	52868.17	52740.17	56045.22	56711.1	57797.51

Figure.6N

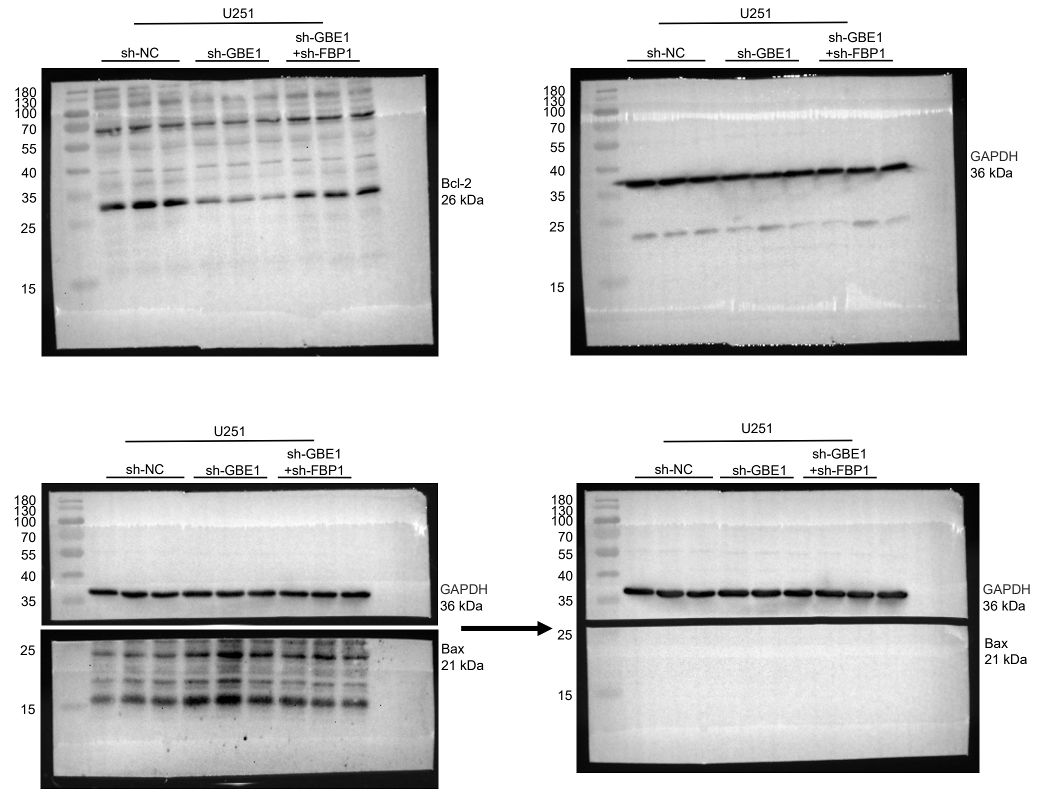

Line	U251 sh-NC	U251 sh-NC	U251 sh-NC	U251 sh-GBE1	U251 sh-GBE1	U251 sh-GBE1	U251 sh-GBE1 +sh-FBP1	U251 sh-GBE1 +sh-FBP1	U251 sh-GBE1 +sh-FBP1
Bcl-2	14912.205	17257.962	13653.033	6551.083	5341.841	3417.376	9104.962	11581.619	13984.619
GAPDH	20695.033	20030.74	17597.912	15625.326	13267.255	18736.811	13288.962	12527.619	13102.912
Bax	6504.962	7083.205	7935.548	12276.447	18476.983	12836.326	11464.912	12555.205	9881.74
GAPDH	19151.033	17735.841	15907.326	16915.276	16361.447	17408.569	19316.154	17876.497	20183.983

Figure.6N

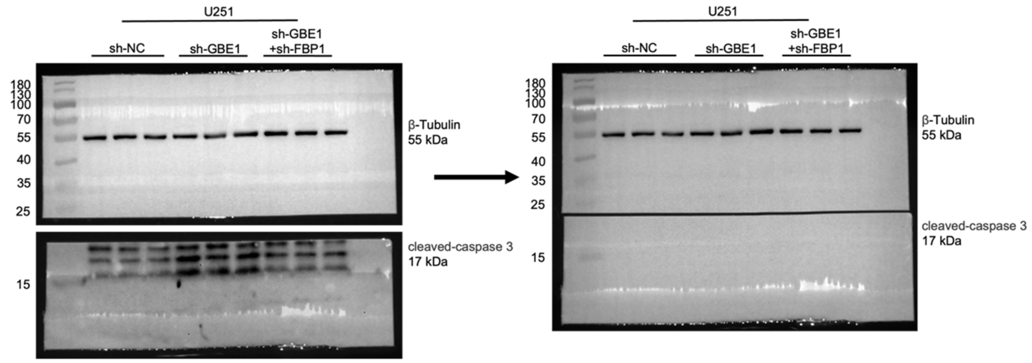

Line	U251 sh-NC	U251 sh-NC	U251 sh-NC	U251 sh-GBE1	U251 sh-GBE1	U251 sh-GBE1	U251 sh-GBE1 +sh-FBP1	U251 sh-GBE1 +sh-FBP1	U251 sh-GBE1 +sh-FBP1
Cleaved-caspase 3	29116.803	26235.631	33775.359	66231.773	54547.137	58255.702	41663.752	33158.217	36380.53
β-Tubulin	62220.51	57458.48	49622.066	48515.338	42461.045	51922.459	58404.217	52861.045	50223.874
